# Charting brain GABA and glutamate levels across psychiatric disorders by quantitative analysis of 121 ^1^H-MRS studies

**DOI:** 10.1017/S0033291724001673

**Published:** 2024-11

**Authors:** Jiayuan Zhang, Timothea Toulopoulou, Qian Li, Lijing Niu, Lanxin Peng, Haowei Dai, Keyin Chen, Xingqin Wang, Ruiwang Huang, Xinhua Wei, Ruibin Zhang

**Affiliations:** 1 Laboratory of Cognitive Control and Brain Healthy, Department of Psychology, School of Public Health, Southern Medical University, Guangzhou, China; 2 Department of Psychology & National Magnetic Resonance Research Center (UMRAM) & Aysel Sabuncu Brain Research Center, Bilkent University, Ankara, Turkey; 3 Department of Psychiatry, National and Kapodistrian University of Athens, Athens, Greece; 4 Department of Psychiatry, Icahn School of Medicine at Mount Sinai, New York, USA; 5 Department of Neurosurgery, Institute of Brain Diseases, Nanfang Hospital of Southern Medical University, Guangzhou, PR China; 6 School of Psychology, South China Normal University, Guangzhou, China; 7 Department of Radiology, Guangzhou First Affiliated Hospital, Guangzhou, PR China; 8 Guangdong-Hong Kong-Macao Greater Bay Area Center for Brain Science and Brain-Inspired Intelligence, Guangdong-Hong Kong Joint Laboratory for Psychiatric Disorders, Guangdong Basic Research Center of Excellence for Integrated Traditional and Western Medicine for Qingzhi Diseases; 9 Department of Psychiatry, Zhujiang Hospital, Southern Medical University, Guangzhou, PR China

**Keywords:** meta-analysis, MRS, neurometabolite, psychiatric disorder

## Abstract

**Background:**

Psychiatric diagnosis is based on categorical diagnostic classification, yet similarities in genetics and clinical features across disorders suggest that these classifications share commonalities in neurobiology, particularly regarding neurotransmitters. Glutamate (Glu) and gamma-aminobutyric acid (GABA), the brain's primary excitatory and inhibitory neurotransmitters, play critical roles in brain function and physiological processes.

**Methods:**

We examined the levels of Glu, combined glutamate and glutamine (Glx), and GABA across psychiatric disorders by pooling data from 121 ^1^H-MRS studies and further divided the sample based on Axis I disorders.

**Results:**

Statistically significant differences in GABA levels were found in the combined psychiatric group compared with healthy controls (Hedge's *g* = −0.112, *p* = 0.008). Further analyses based on brain regions showed that brain GABA levels significantly differed across Axis I disorders and controls in the parieto-occipital cortex (Hedge's *g* = 0.277, *p* = 0.019). Furthermore, GABA levels were reduced in affective disorders in the occipital cortex (Hedge's *g* = −0.468, *p* = 0.043). Reductions in Glx levels were found in neurodevelopmental disorders (Hedge's *g* = −0.287, *p* = 0.022). Analysis focusing on brain regions suggested that Glx levels decreased in the frontal cortex (Hedge's *g* = −0.226, *p* = 0.025), and the reduction of Glu levels in patients with affective disorders in the frontal cortex is marginally significant (Hedge's *g* = −0.172, *p* = 0.052). When analyzing the anterior cingulate cortex and prefrontal cortex separately, reductions were only found in GABA levels in the former (Hedge's *g* = − 0.191, *p* = 0.009) across all disorders.

**Conclusions:**

Altered glutamatergic and GABAergic metabolites were found across psychiatric disorders, indicating shared dysfunction. We found reduced GABA levels across psychiatric disorders and lower Glu levels in affective disorders. These results highlight the significance of GABA and Glu in psychiatric etiology and partially support rethinking current diagnostic categories.

## Introduction

Currently, psychiatric diagnosis primarily relies on categorical classification systems, such as DSM-5, which delineate distinct disorders; however, clinical characteristics and genetic factors often cut across these diagnostic boundaries (Smoller et al., 2019). Current clinical diagnostic criteria, which allow for categorizing mental disorders into neurodevelopmental, affective, and psychotic, are primarily based on clinical and behavioral manifestations (Bagby, Uliaszek, Gralnick, & Al-Dajani, 2017), and they are at odds with observations of comorbidity across disorders. For instance, attention deficit hyperactivity disorder (ADHD) frequently coexists with other disorders, including autism spectrum disorder (ASD) (Kadesjö & Gillberg, 2001) and bipolar disorder (BD) (Faraone, 2018; Tamam, Karakus, & Ozpoyraz, 2008), and it shares genetic, social, and neurobiological characteristics with them (Anticevic et al., 2015). Similarly, there is significant comorbidity between major depressive disorder (MDD) and schizophrenia (Siris, 2000; Siu, Chong, & Lo, 2018). The Research Domain Criteria (RDoC) framework offers a novel classification system based on observable behavior dimensions and neurobiological measures to provide biopsychological interpretations of clinical issues (Kozak & Cuthbert, 2016) and a more comprehensive understanding of traits shared among various psychiatric disorders.

The biological hypothesis for psychiatric disorders is primarily focused on neurotransmitter systems (Blokhin, Khorkova, Saveanu, & Wahlestedt, 2020). Glutamate (Glu) is the primary excitatory neurotransmitter, and gamma-aminobutyric acid (GABA) is the brain's primary inhibitory neurotransmitter. Glu is crucial for brain functionality and development, and it binds to *N*-methyl-d-aspartate receptors (NMDARs), which are critical for neuronal communication (Paoletti, Bellone, & Zhou, 2013; Zhu et al., 2018). Glutamatergic dysfunction has been associated with several psychiatric disorders, including MDD (Hashimoto, 2009; Sanacora, Treccani, & Popoli, 2012), ASD (Nisar et al., 2022), and schizophrenia (Egerton et al., 2020). Evidence supporting Glu's role in psychiatric disease includes the synergistic interaction between monoamines and antidepressants, elevated prefrontal Glu concentrations in MDD, dysregulated modulation of striatal dopamine neurotransmission and NMDAR hypofunction in schizophrenia (Balu, 2016; Kruse & Bustillo, 2022; Nakazawa & Sapkota, 2020), and altered gut-microbiota-mediated Glu metabolism noted in ASD (Montanari, Martella, Bonsi, & Meringolo, 2022). Therefore, glutamatergic dysregulation may affect the pathophysiology of various psychiatric disorders. GABA, however, plays a crucial role in neural-network dynamics via GABAergic interneurons. Beyond its structural contributions, GABA has instrumental roles in physiological processes such as neural plasticity and stress reactivity. GABAergic inhibition plays a pivotal role in the pathogenesis of neurodevelopmental disorders (Tang, Jaenisch, & Sur, 2021) and psychotic disorders (Zahid et al., 2023). GABAergic dysfunction, particularly reduced signaling through the GABA_A_ receptor pathway, has been implicated in the pathophysiology of a wide range of psychiatric disorders, contributing significantly to cognitive impairments (Prévot & Sibille, 2021; Zhu et al., 2018).

Previous animal studies have linked depression to GABA deficit and reduced GABA_A_ receptor activity (Belozertseva & Andreev, 1997; Kram, Kramer, Steciuk, Ronan, & Petty, 2000), and genetic models have highlighted the role of decreased NMDAR activity in synaptic plasticity and neuronal survival. Further studies have associated Glu and GABA receptor activity with homeostatic regulation of neuronal excitation (Wen et al., 2022). An imbalance between excitatory pyramidal neurons and inhibitory interneurons during neurodevelopment may trigger localized Glu surges, leading to excessive dendritic pruning, activating local dendritic apoptosis, and contributing to psychotic disorders (Parellada & Gassó, 2021). Animal models of various disorders (e.g. schizophrenia and ASD) suggest shared mechanisms in disease pathogenesis, including neurotransmitter involvement (Nestler & Hyman, 2010; Robbins, Vaghi, & Banca, 2019); however, these models often base their findings on specific etiological factors, which means that there is a lack of models for idiopathic disorders, among other problems (Palmer et al., 2019). *In vivo* human studies have a comparative advantage in terms of accurately representing human biology and disease processes.

Proton magnetic resonance spectroscopy (^1^H-MRS) is a non-invasive technique that allows *in vivo* measurement of brain metabolites without administering radioactive tracers or drugs (Rothman et al., 2019). For various psychiatric disorders, advances in ^1^H-MRS techniques and higher-field MRI scanners have revealed significant alterations in neurotransmitter levels in specific brain regions. For instance, it has been found that patients with psychotic disorders exhibit reduced GABA levels in the dorsal anterior cingulate cortex (ACC) and elevated Glu levels in the thalamus (Thal) (Bojesen et al., 2020). Studies of affective disorders have reported decreased GABA levels in the ACC and the medial prefrontal cortex (PFC) (Li et al., 2016), but elevated GABA levels in the PFC (Draganov et al., 2020). Neurodevelopmental disorders have been found to be associated with reduced GABAergic levels in the ACC, decreased Glu in the striatum, and altered glutamatergic and GABAergic transmission in the left cerebellum (Horder et al., 2018; Ito et al., 2017). However, whether these alterations are common across disorders or specific to each condition remains unclear. Meta-analysis offers a valuable approach to integrating these diverse results and reconciling their inconsistencies.

Existing meta-analyses have revealed diminished brain GABA levels in patients with ASD and MDD (Schür et al., 2016), with similar deficiencies observed in the frontal cortex and ACC of individuals with schizophrenia (Kumar, Vajawat, & Rao, 2021). Glutamatergic metabolites are principally reduced in the ACC in MDD, while Glx and glutamine (Gln) are augmented in the ACC in cases of BD (Li et al., 2016; Luykx et al., 2012). Some research has concurrently examined GABAergic and glutamatergic metabolites. For example, it was found that in MDD, cortical GABA and Glx levels were diminished in the ACC (Godfrey, Gardner, Kwon, Chea, & Muthukumaraswamy, 2018); in schizophrenia, GABA levels in the midcingulate cortex were depleted, while Glx and Glu levels were elevated (Nakahara et al., 2022). The results for each brain region in these studies were found to be relatively dispersed. Given the inherently limited spatial resolution of H^1^-MRS imaging and the overlap among some brain regions, it is reasonable to combine the regions to allow for a more focused observation of effects. Overall, previous meta-analyses have shown varying trends in GABA and Glu in various psychiatric disorders across multiple brain areas (see Table 1 for a summary of previous meta-analyses), but these studies mostly focused on a single psychiatric disorder or scrutinized a solitary metabolite across several disorders. Few studies have considered both GABA and Glu to determine whether these alterations in excitatory and inhibitory metabolites are consistent across psychiatric studies or examined the balance between these two metabolites across psychiatric disorders (Kubota, Moriguchi, Takahata, Nakajima, & Horita, 2020; Schür et al., 2016; Truong et al., 2021). As such, there remains a notable research gap in terms of studies exploring both metabolites across various disorders.

**Table 1.** Main findings of the existed meta-analysis characterizing the GABA Glu and Glx in psychiatric disorders

Study	Measurement	Characteristics and sample	Main findings
Luykx et al. (2012)	Glu, Glx	Glu: 7 MDD studies, with 156 patients and 160 controls; Glx: 13 MDD studies, with 224 patients and 237 controls.	**Glu** **ACC:**−0.86[−1.55, −0.17]↓. **Glx** **ACC:**−1.15[−1.86, −0.44]↓; **Whole brain:**−0.62[−1.17, −0.07]↓.
Schür et al. (2016)	GABA	GABA: 13 MDD studies, 10 SZ studies, 5 ASD studies, 4 BD studies, 3 PD studies, 3 PTSD studies, 3 ADHD studies.	**GABA (Whole brain)** **ASD:** −0.74[−1.171, −0.307]↓; **MDD: −**0.52[−0.885, −0.156]↓.
Merritt et al. (2016)	Glu, Glx	59 SZ studies, with 1686 patients and 1451 controls.	**Glx** **Mfc:** *g* = 0.26[0.05, 0.46]↑; **Frontal white matter:** *g* = 0.42[0.18, 0.66]↑; **MTL:** *g* = 0.32[0.12, 0.52]↑; **Basal ganglia:** *g* = 0.39[0.09, 0.70]↑. **Glu** **Basal ganglia:** *g* = 0.63[0.15, 1.11]↑. **Gln** **Thalamus:** *g* = 0.56[0.02; 1.09]↑.
Godfrey et al. (2018)	GABA, Glu, Glx	GABA: 18 MDD studies, with 356 patients and 366 healthy controls. Glx: 24 MDD studies, with MDD patients and 501 controls. Glu:18 MDD studies, with 444 patients and 420 healthy controls.	**GABA** **Whole brain:**−0.35 [−0.61, −0.10]↓; **ACC:**−0.56 [−0.93, −0.18]↓. **Glx** **ACC:**−0.40 [−0.81; −0.01]↓.
Nakahara et al. (2022)	GABA, Glu, Gln, Glx	GABA: 30 SZ studies. Glu: 68 SZ studies. Gln: 11 SZ studies. Glx: 84 SZ studies.	**GABA** **MCC:** *g* = −0.40[−0.62, −0.17]↓. **Glu** **MCC:** *g* = −0.17[ −0.33, −0.01]↓. **Glx** **Basal ganglia:** *g* = 0.32[0.18, 0.45]↑; **Hippocampus:** *g* = 0.47[0.21, 0.73]↑; **Dpfc:** *g* = 0.25[0.05, 0.44]↑.

PD, panic disorder; PTSD, posttraumatic stress disorder; *g*, Hedge's *g*. Dpfc, Dorsolateral prefrontal cortex; ↑, indicates increasing; ↓, indicates decreasing. Main findings included significant effect size with 95% CIs in patients *v*. controls

To address this research gap, we conducted a three-step meta-analysis of ^1^H-MRS studies examining GABA and Glu levels. First, we studied overall neurometabolite levels in all patients, disregarding specific diagnoses and brain regions. Second, we examined individual disorders separately within the Axis I classification. Finally, we performed subanalyses on the results of the first two steps to examine neurometabolites within specific brain regions and diagnostic classifications. As there are numerous factors that could modulate the accuracy or precision of findings (e.g. antipsychotic drugs or anxiolytics), we considered important moderators in our analysis, including medication status and magnetic-field strength. Based on the majority of ^1^H-MRS studies, we hypothesized that there are GABA and Glu reductions across psychiatric disorders.

## Methods

### Search methods for identification of studies

We systematically searched PubMed and Web of Science for studies that employed ^1^H-MRS to report on the levels of GABA, Glu, or Glx in individuals with psychiatric disorders compared to healthy controls (HCs) on 20 July 2022. The following search terms were used: ‘MRS’ or ‘Spectroscopy’ or ‘Spectroscopic’ or ‘Mrsi’ or ‘(Proton) Magnetic Resonance Spectroscopy’ or ‘(1H)MRS’ and ‘GABA’ or ‘Gamma-aminobutyric acid’ or ‘Gamma aminobutyric acid’ or ‘GABAergic’ or ‘Brain Metabolites’ or ‘Brain Biochemistry’ or ‘Glutamate’ or ‘Glutamine’ or ‘Glutamic Acid’ or ‘Excitatory Amino Acid’ or ‘inhibitory Amino Acid’, and ‘Psychiatry’ or ‘Psychiatric’ or ‘MDD’ or ‘Major depressive disorder’ or ‘Mental’ or ‘Bipolar’ or ‘ASD’ or ‘Autism spectrum disorder’ or ‘Disorder’ or ‘Panic’ or ‘ADHD’ or ‘Attention deficit and hyperactivity disorder’ or ‘Schizophrenia’ or ‘Anxiety’ or ‘Dependence ‘ or ‘Schizophrenic’ or ‘Phobia’ or ‘Addiction’ or ‘Depression’ or ‘PTSD’ or ‘Post-traumatic stress disorder’ or ‘Nervosa’. The reference lists of retrieved articles were also screened for further relevant studies.

### Criteria for considering studies for this review

**Figure 1.**
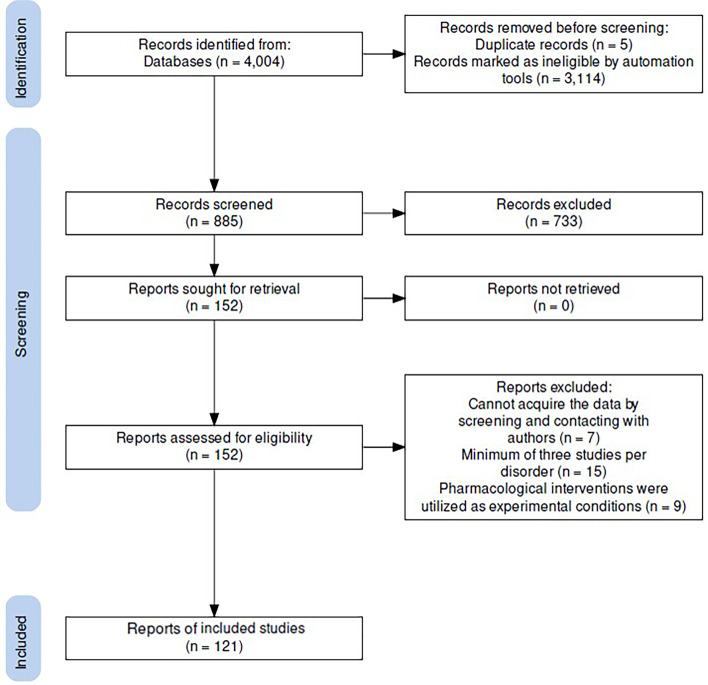
PRISMA diagram of the literature search.

Based on titles and abstracts, we meticulously selected articles for our examination based on the following criteria: (1) *in vivo* human ^1^H-MRS studies for the level of metabolite; (2) using editing-technique or J-resolved ^1^H-MRS to measure GABA or Glu (includes Glx); (3) included comparison with HCs; (4) patients with severe substance abuse were excluded; (5) original article; (6) article reported using English. Full-text reviews of the selected studies were conducted to verify inclusion criteria (Fig. 1). Following similar meta-analyses (Du et al., 2023; Luykx et al., 2012; Valentine, Pigott, & Rothstein, 2010), a minimum of 3 studies per psychiatric category was required to be included in the current meta-analysis; this is because such small numbers of studies would mean that the number and scope of significant research findings were limited.

### Data extraction

The extracted data included: the means and standard deviations (SDs) of GABA, Glu, and Glx levels; sample sizes; selected brain regions; and demographic/clinical characteristics (e.g. gender, age, diagnosis, and psychotropic medication use). The regions of interest were categorized into the following: (1) frontal cortex, including ACC and PFC due to the spatial overlap of these voxels; (2) occipital cortex; (3) parieto-occipital cortex; (4) thalamus; (5) striatum; (6) basal ganglia (including caudate and putamen); (7) hippocampus; (8) temporal cortex (Temp; including middle temporal area, MT).

The ^1^H-MRS methodology details included: magnetic-field strength, specific editing technique or J-resolved ^1^H-MRS, and metabolite quantification using water or creatine as references. The medication-usage status of patient cohorts included the proportions of psychotropic medication and durations of discontinuation. In situations in which a study provided data from multiple brain regions, we incorporated these as distinct samples, segmented by their respective regions, for integration into the analysis. Duplicate data from the same samples reported in different publications were included based on the publication with the largest sample size. We categorized the collected disorders by Axis I into neurodevelopmental disorders (including ADHD and ASD, *N* = 25 studies), affective disorders (including MDD and BD, *N* = 61 studies), psychotic disorders (including schizophrenia, *N* = 28 studies). Other psychiatric disorders included alcohol use disorder (AUD; *N* = 7 studies). AUD was chosen without including substance-abuse disorder for two reasons: first, due to the insufficient number of studies on substance addiction, and second, because the wide ranges of drug intake in substance addiction are not conducive to exploring the mechanisms of neurotransmitters.

### Statistical analysis

For the primary analyses, we examined the overall levels of specific neurometabolites across all patients regardless of distinct diagnoses and brain regions. In the secondary analyses, we considered neurometabolites separately for each disorder based on its Axis I classification. We also examined brain regions separately. The effect sizes of the included studies were separately calculated using sample sizes, mean neurometabolite levels, and SDs. If the SDs were unavailable, sample sizes and *p* values were employed to calculate Hedges' *g*. The effect size was computed as the standardized mean difference in brain-metabolite levels between patients and HCs, converted to Hedges' *g* with a 95% confidence interval (CI). A random-effects model was used to account for potential biases resulting from sample sizes. We compared the effect sizes in three broad classes of disorders – affective disorders (e.g. MDD and BD combined), neurodevelopmental disorders (ADHD and ASD combined), and psychotic disorders – and separately for each available Axis I disorder (e.g. ADHD, schizophrenia). Finally, we performed two sets of subanalyses to further investigate our findings. The first set involved grouping the data based on brain regions, and the second set involved categorizing the data based on (1) diagnostic classifications according to the DSM and (2) brain regions. In cases of multiple regions having been analyzed within a single study, we analyzed concentration levels separately instead of averaging them. We also assumed common/varied variance components across subgroups among studies and integrated the subgroups using a random-effects model.

Heterogeneity was evaluated using Cochrane's *Q* test and the *I*^2^ statistic; *I*^2^ scores greater than 25, 50, and 75% were defined as corresponding to low, moderate, and high heterogeneity, respectively, and *I*^2^ scores <25% were considered acceptable (Higgins, Thompson, Deeks, & Altman, 2003). Funnel plots were constructed, and Egger's test was used to establish possible publication bias (Egger, Davey Smith, Schneider, & Minder, 1997). The potential publication bias was evaluated using Egger's test, with *p* values less than 0.05 suggesting significant publication bias. Sensitivity analysis was conducted using leave-one-out analysis, and visual inspection of funnel plots was performed to further ascertain any potential publication bias. To further investigate the heterogeneity of brain regions, a variability analysis using the log coefficient of variation ratio (CVR) was conducted (Merritt et al., 2023). A CVR value above 0 was taken to signify increased variability among patients, whereas a value below 0 was taken to indicate heightened variability within controls.

### Moderator analysis

We employed a meta-regression analysis to investigate the potential impact of four key demographic and clinical variables on the metabolite levels: sex, age, magnetic-field strength, and psychoactive medication use (Med) (Befroy & Shulman, 2011; Tkác, Oz, Adriany, Uğurbil, & Gruetter, 2009). To further explore the impact of magnetic-field strength on neurotransmitter levels, we conducted a subgroup analysis at different magnetic-field strengths (specifically 1.5, 3.0, 4.0 and 7.0 T). The mean age was calculated for both patients and HCs to predict metabolite levels. In recognition of the potential effects of psychotropic medication on psychiatric patients, we defined medication status using the proportions of patients on psychotropic medication and the durations of medication discontinuation.

Discontinuation periods were categorized as follows: current treatment, short-term (discontinuation duration less than six months), long-term (discontinuation duration more than six months), and never received treatment. We then used these medication-status classifications to predict the levels of metabolites. All analyses were executed using RStudio and the Comprehensive Meta-Analysis software developed by Biostat (Borenstein, Hedges, Higgins, & Rothstein, 2009).

## Results

### General characteristics of the included studies

A total of 121 studies that met the criteria were included in this meta-analysis (Fig. 1, Table 2, and online Supplementary Table S1 in the Supplementary Material). Patient ages ranged from 10.2 to 72.7 years old. A total of thirteen (10.7%) studies were performed at 7.0 T, eighty-five (70.2%) studies were performed at a magnetic-field strength of 3.0 T, seven (5.8%) at 4.0 T, and nine (7.4%) studies were performed at 1.5 T (online Supplementary Table S1). Detailed information pertaining to the ^1^H-MRS protocols used and the measurement parameters, including the number of studies, brain regions, and sample sizes for the primary and secondary analysis, are outlined in Table 2.

**Table 2.** Summary of comparative outcomes for measurements

Metabolites	Region	Studies	Patients/Control	Sample size	Hedge's *g* (95% *CI*)	*p* for Hedge's *g*	*I*^2^	*p* for heterogeneity
*Affective disorder*								
GABA								
	ACC	12	322/263	585	−0.155 (−0.480 to 0.170)	0.349	71.630	<0.001
	**OCC**	**11**	**198/195**	**383**	**−468 (−0.921 to −0.015)**	**0.043**	**72.250**	**<0.001**
	PFC	6	112/123	340	0.072 (−0.242 to 0.386)	0.652	50.22	0.041
	Total	32	712/721	1701	−0.148 (−0.334 to 0.038)	0.119	69.741	<0.001
Glu								
	**ACC**	**18**	**534/479**	**1013**	**−0.281 (−0.525 to −0.37)**	**0.024**	**67.620**	**<0.001**
	OCC	8	177/161	344	0.03 (−0.226 to 0.231)	0.981	0	0.993
	PFC	11	214/257	475	−0.012 (−0.218 to 0.194)	0.909	17.816	0.274
	Total	36	920/906	2373	−0.046 (−0.227 to 0.134)	0.614	76.400	<0.001
Glx								
	ACC	10	147/159	346	−0.448(−1.006 to 0.111)	0.116	82.690	<0.001
	OCC	5	59/79	148	0.969 (−0.052 to 1.990)	0.063	88.480	<0.001
	PFC	13	145/197	474	−0.333 (−0.558 to −0.108)	**0.004**	28.850	0.155
	Total	25	593/624	1542	−0.096 (−0.320 to 0.128)	0.4	77.040	<0.001
*Neurodevelopmental disorder*								
GABA								
	ACC	7	226/186	492	0.232 (−0.488 to 0.024)	0.076	44.502	0.094
	OCC	3	74/83	151	0.012 (−0.342 to 0.366)	0.948	0	0.404
	PFC	8	188/193	336	0127 (−0.302 to 0.556)	0.562	73.207	<0.001
	Total	24	610/611	2149	−0.132 (−0.278 to 0.014)	0.076	65.125	<0.001
Glu								
	ACC	5	192/152	344	−0.074 (−0.290 to 0.143)	0.505	0	0.8
	PFC	3	55/54	131	0.652 (−0.392 to 1.697)	0.221	87.944	<0.001
	Total	9	263/225	689	0.180 (−0.241 to 0.601)	0.403	78.141	<0.001
Glx								
	ACC	5	192/143	415	−0.548 (−1.188 to 0.092)	0.093	88.646	<0.001
	PFC	5	119/122	239	−0.123 (−0.495 to 0.249)	0.517	80.578	<0.001
	**Total**	**16**	**498/458**	**1678**	**−0.292 (−0.542 to −0.043)**	**0.022**	**83.017**	**<0.001**
*Psychotic disorder*								
GABA								
	**ACC**	**13**	**331/499**	**927**	**−0.215 (−0.427 to −0.003)**	**0.047**	**47.204**	**0.030**
	PFC	8	243/264	576	0.174 (−0.209 to 0.558)	0.372	80.179	<0.001
	Total	22	722/901	3160	−0.045 (−0.21 to 0.121)	0.596	68.207	<0.001
Glu								
	ACC	8	259/280	539	−0.107 (−0.432 to 0.218)	0.519	69.185	0.002
	PFC	6	232/252	484	−0.070 (−0.406 to 0.267)	0.685	68.279	0.008
	Total	17	718/759	2509	−0.005 (−0.150 to 0.141)	0.950	68.726	<0.001
Glx								
	ACC	7	206/222	428	0.105 (−0.423 to 0.633)	0.696	83.973	<0.001
	PFC	4	137/154	325	0.043 (−0.743 to 0.829)	0.914	90.543	<0.001
	Total	13	471/475	1996	0.077 (−0.101 to 0.255)	0.396	72.612	<0.001

The bolded values represent statistically significant results for Hedge's *g*.

### Brain GABA levels

#### All patients regardless of diagnosis

**Figure 2.**
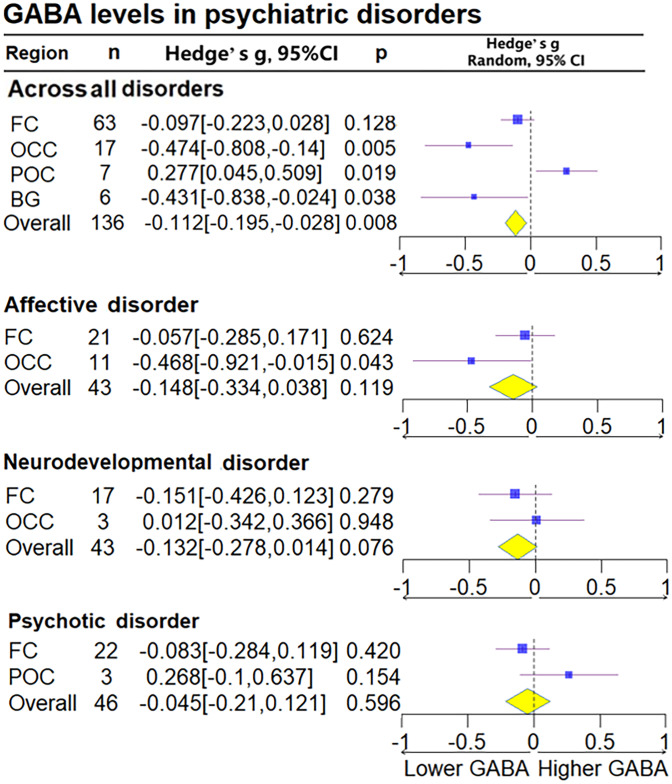
Subgroup forest plot of GABA levels. FC, frontal cortex; BG, basal ganglia; POC, parieto-occipital cortex; Thal, thalamus cortex; Temp, temporal cortex; OCC, occipital cortex. Overall, all studies levels without dividing subgroup. Lower GABA, represent the lower relative deviation from zero as the standard reference point, indicates that the GABA concentration in the patient group is lower than that in the control group when comparing the two populations; Higher GABA, represent the higher relative deviation from zero as the standard reference point. Diamond shaped yellow symbols represent the overall effect. Blue squares represent the subgroup effect and the size is proportionate to the sample size used for each region.

We found GABA levels to be reduced in the pooled patient sample compared to HCs, regardless of diagnosis (Hedge's *g* = −0.112, 95% CI −0.195 to −0.028, *p* = 0.008). Subanalysis based on the regions of interest indicated that all patients exhibited significantly lower GABA levels in the ACC (Hedge's *g* = −0.191, 95% CI −0.334 to −0.049, *p* = 0.009), basal ganglia (Hedge's *g* = −0.431, 95% CI−0.838 to − 0.024, *p* = 0.038), and OCC (Hedge's *g* = −0.478, 95% CI −0.808 to −0.141, *p* = 0.005), but not in the FC (Hedge's *g* = −0.097, 95% CI −0.223 to 0.028, *p* = 0.128) after combining the PFC and ACC compared with HCs. All patients exhibited significantly higher levels in the POC (Hedge's *g* = 0.276, 95% CI 0.049–0.503, *p* = 0.017) compared with HCs (Fig. 2 and online Supplementary Fig. S2 in the Supplementary Material).

#### Affective disorders

Overall GABA levels in patients with affective disorders were not significantly different (Hedge's *g* = −0.148, 95% CI −0.334 to 0.38, *p* = 0.119) (online Supplementary Fig. S1 in the Supplementary Material). Subanalysis based on brain regions, however, indicated lower GABA levels in the OCC (Hedge's *g* = −0.468, 95% CI −0.921 to −0.015, *p* = 0.043). Further analysis examining affective disorders separately suggested lower GABA levels compared to HCs only in MDD (Hedge's *g* = −0.305, 95% CI −0.534 to −0.075, *p* = 0.009) (Fig. 2 and online Supplementary Fig. S3 in the Supplementary Material).

#### Neurodevelopmental disorders

No statistically significant differences in GABA levels were found in neurodevelopmental disorders (Hedge's *g* = −0.132, 95% CI −0.278 to 0.014, *p* = 0.076), including when we examined these separately in ADHD and ASD. Subanalysis based on brain regions in neurodevelopmental disorders also yielded no statistically significant differences (online Supplementary Figs S3 and S4 in the Supplementary Material).

#### Psychotic disorders

No statistically significant differences in GABA levels were found in patients with psychotic disorders (Hedge's *g* = −0.045, 95% CI −0.21 to 0.121, *p* = 0.596) (online Supplementary Fig. S4 in the Supplementary Material). In subgroup analysis based on brain regions, patients with schizophrenia exhibited significantly reduced GABA levels in the ACC (Hedge's *g* = −0.215, 95% CI −0.427 to −0.003, *p* = 0.047) but not in the FC (Hedge's *g* = −0.083, 95% CI −0.284 to 0.119, *p* = 0.42) (online Supplementary Figs S2 and S5 in the Supplementary Material).

### Brain Glu and Glx levels

#### All patients regardless of diagnosis

**Figure 3.**
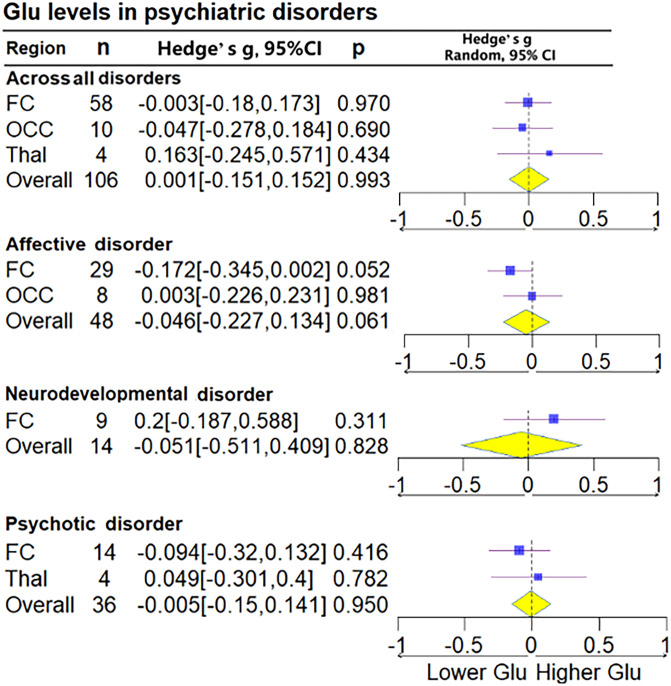
Subgroup forest plot of Glu levels. Lower Glu, represent the lower relative deviation from zero as the standard reference point, indicates that the Glu concentration in the patient group is lower than that in the control group when comparing the two populations; Higher Glu, represent the higher relative deviation from zero as the standard reference point.

**Figure 4.**
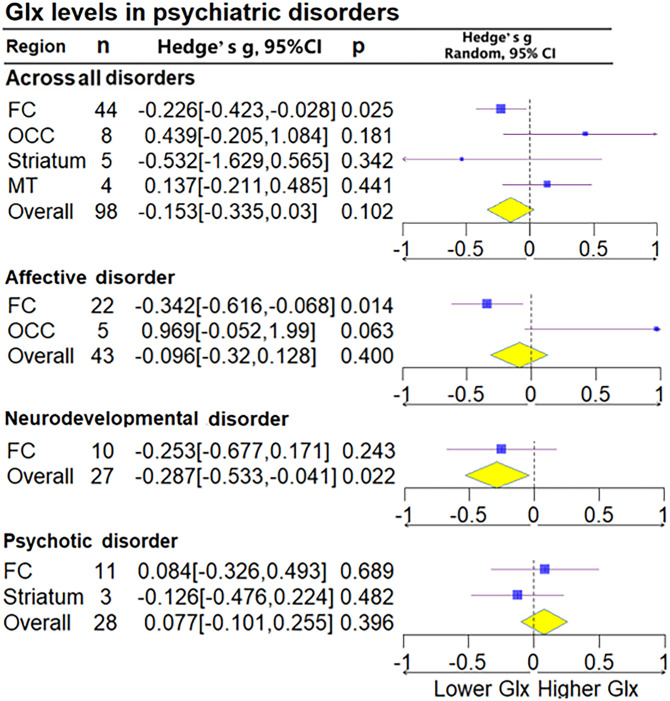
Subgroup forest plot of Glx levels. Lower Glx, represent the lower relative deviation from zero as the standard reference point, indicates that the Glx concentration in the patient group is lower than that in the control group when comparing the two populations; Higher Glx, represent the higher relative deviation from zero as the standard reference point.

Glu for all brain regions together was measured in 106 patient groups and 106 control groups (68 studies), and Glx for all brain regions together was measured in 98 cases and 98 controls (51 studies). No statistically significant differences in Glu (Hedge's *g* = −0.001, 95% CI −0.151 to 0.152, *p* = 0.993) or Glx (Hedge's *g* = −0.153, 95% CI −0.335 to 0.030, *p* = 0.102) were found. Subgroup analysis of brain regions found decreases in Glx levels in the FC (Hedge's *g* = −0.226, 95% CI −0.423 to 0.028, *p* = 0.025) but not in Glu levels (Hedge's *g* = −0.003, 95% CI −0.18 to 0.173, *p* = 0.97) (Figs 3 and 4).

#### Affective disorders

Patients with affective disorders did not show significantly different Glu levels compared with HCs (Hedge's *g* = −0.148, 95% CI −0.334 to 0.038, *p* = 0.119) (Fig. 3). Glx levels in patients with affective disorders exhibited significance in the FC (Hedge's *g* =  −0.342, 95% CI −0.616 to −0.068, *p* = 0.014), whereas Glu levels showed marginal significance (Hedge's *g* = −0.172, 95% CI −0.345 to 0.002, *p* = 0.052). Analysis conducted separately on the ACC and PFC within the FC found that patients with affective disorders exhibited decreased Glu levels in the ACC (Hedge's *g* = −0.281, 95% CI −0.525 to −0.037, *p* = 0.024). Furthermore, Glx levels were significantly lower in the PFC (Hedge's *g* = −0.333, 95% CI −0.558 to −0.108, *p* = 0.004). Upon conducting a subgroup analysis based on diagnosis, patients with MDD and BD exhibited no significant difference in Glu and Glx levels (online Supplementary Figs S6–S9 in the Supplementary Material).

#### Neurodevelopmental disorders

Patients with neurodevelopmental disorders were found to have significantly lower Glx levels (Hedge's *g* = −0.287, 95% CI −0.533 to 0.031, *p* = 0.022) (online Supplementary Fig. S7 in the Supplementary Material). Analysis based on brain regions did not indicate significant differences. In separate analyses of ADHD and ASD, patients with ADHD were found to exhibit no significant differences in Glu and Glx levels, and those with ASD also did not exhibit significant differences in Glu and Glx levels (online Supplementary Figs S10 and S11 in the Supplementary Material).

#### Psychotic disorders

No statistically significant differences in Glu (Hedge's *g* = −0.005, 95% CI −0.150 to 0.141, *p* = 0.950) and Glx levels (Hedge's *g* = 0.077, 95% CI −0.101 to 0.255, *p* = 0.396) were found between patients with schizophrenia and HCs, including in the subgroup analysis divided by brain regions (Figs 3 and 4).

### Meta-regression

#### Effect of age on GABA, Glu, and Glx

Hedge's *g* did not significantly depend on age for GABA (*R*^2^ = 0.00, *p* = 0.527), Glu (*R*^2^ = 0.00, *p* = 0.226), or Glx levels (*R*^2^ = 0.00, *p* = 0.399), suggesting that the levels of GABA, Glu, and Glx levels do not exhibit alterations in correlation with age-related changes (online Supplementary Table S2).

#### Effect of medication use

The medication status had no statistically detectable moderating effect on GABA, Glu, or Glx. The durations of medication discontinuation also had no effect on these neurotransmitters (online Supplementary Table S2).

#### Effect of ^1^H-MRS methodology

The methodological ^1^H-MRS parameters varied widely across studies, and we mainly considered the editing technique and MRI field strength. Regarding the editing technique, 32 studies used MEGA-PRESS and 18 studies used an in-house editing technique (J-editing, JPRESS, MEGA-sLASER, or J-resolved MRS). Creatine was used as a reference compound in 45 studies, water (H_2_O) in 11 studies, and three studies reported values for both. We analyzed data for 1.5, 3.0, 4.0, and 7.0 T field strengths to investigate potential differences in detected concentrations dependent upon field strength. Hedge's *g* did not significantly depend on field strength for GABA (*R*^2^ = 0.00, *p* = 0.396), Glu (*R*^2^ = 0.06, *p* = 0.114), or Glx levels (*R*^2^ = 0.00, *p* = 0.696) (online Supplementary Table S2). Furthermore, we conducted subgroup analysis for field strength and found that Glx levels were lower at both 1.5 T (Hedge's *g* − 0.999, 95% CI −1.801 to −0.197, *p* = 0.015) and 7 T (Hedge's *g* = −0.255, 95% CI −0.428 to −0.083, *p* = 0.004), GABA levels were lower at 3 T (Hedge's *g* = −0.151, 95% CI −0.280 to −0.022, *p* = 0.022), and Glu levels were lower at 7 T (Hedge's *g* = 0.202, 95% CI −0.349 to −0.055, *p* = 0.007) (online Supplementary Fig. S12 in the Supplementary Material).

### Heterogeneity and publication bias

Significant heterogeneity persisted across GABA, Glu, and Glx studies within psychotic, affective, and neurodevelopmental disorders, with *I*^2^ values ranging from 48.69% to 87.85% (*p* < 0.001). No significant differences were observed in Glx levels for BD, although with a low heterogeneity (*p* = 0.122, *I*^2^ = 38.65%). Excluding the study with the highest Hedge's *g* value slightly reduced but did not eliminate heterogeneity across all metabolites. In comparison to control subjects, patients exhibited increased variability in GABA levels in the OCC (CVR = 0.237, *p* = 0.0251), in Glu levels in the Thal (CVR = 0.217, *p* = 0.010), and in Glx levels in the FC (CVR = 0.231, *p* < 0.001) (online Supplementary Fig. S13).

Funnel plots and Egger's tests indicated publication bias for Glu in neurodevelopmental disorders and Glx in psychotic disorders. GABA showed significance only for neurodevelopmental disorders (two-tailed *p* = 0.035), while Glu and Glx were significant for psychotic (two-tailed *p* = 0.034) and neurodevelopmental disorders (two-tailed *p* = 0.008), respectively. Other results demonstrated Hedge's *g* values that were symmetrically distributed around the mean, corresponding to higher standard errors (online Supplementary Figs S14–S16 in the Supplementary Material).

## Discussion

This meta-analysis examined 121 studies to investigate metabolite levels measured using ^1^H-MRS in different psychiatric disorders. Overall brain GABA levels were found to be significantly lower in the combined patient sample, and they were specifically diminished within the ACCs of patients with affective or psychotic disorders. Conversely, examination of glutamatergic metabolites revealed significant reductions in Glu levels within the ACCs of patients with affective disorders and significant declines in Glx levels across neurodevelopmental disorders in aggregate and within the PFCs of patients with affective disorders. The results of separate analyses in neurodevelopmental disorders for ASD and ADHD were in line with those of previous studies, which have suggested that cerebellum metabolite levels do not differentiate these groups (Pang et al., 2023). However, in affective disorders, differences in metabolite levels exist, and these may not align with the current classification system that combines MDD and BD, highlighting the heterogeneity and complexity of neurochemical dysregulation in different psychiatric disorders. More importantly, the trends in metabolite levels indicate a decrease across neurodevelopmental disorders, affective disorders, and psychotic disorders. These findings suggest that there may be no significant differences in the Axis I classifications of these disorders, supporting the notion that there are overlapping features and common underlying mechanisms that transcend specific psychiatric diagnoses.

Given the substantial anatomical overlap between the ACC and the PFC, delineating these regions with precision in neuroanatomical studies poses a significant challenge. This overlap complicates the detection of differential effects across the brain's extensively dispersed subregions. In response, we opted for an integrated reanalysis of the ACC and PFC regions. For instance, the significance of Glu in the ACC for affective disorders and GABA in the ACC for psychotic disorders diminished after separate analysis. However, combining these two regions into a larger, unified area may lead to results that are less precise. For instance, after combining, the overall significance of Glx in the FC became significant. Therefore, for reference, we have provided the outcomes of the brain regions before their amalgamation, enabling a more thorough comparison.

### Decreased brain GABA levels across psychiatric disorders

Alterations in GABA metabolism are crucial for regulating cortical excitability (Petroff, 2002) and play a vital role in brain function and physiological processes. Our main finding is that brain GABA levels are lower in those with psychiatric disorders compared with HCs, demonstrating a prevalent GABA imbalance. This result is broadly in line with the existing literature. Differences in GABA levels have nonetheless been reported between different brain regions, and our regional analysis suggested two key findings: lower GABA levels in the ACC and higher levels in the POC, aligning with several studies showing that GABA alterations play a role in the etiology of mental illness (Kolodny, Schallmo, Gerdts, Bernier, & Murray, 2020; Wijtenburg et al., 2021). GABA is considered an important regulatory metabolite in the ACC when reductions are associated with impulsivity, aggression, and possible distraction (Comai, Tau, & Gobbi, 2012; Ende et al., 2016). Elevated brain GABA levels in the POC, which is functionally linked through the default mode network, may be associated with psychotic disorders (Ongür, Prescot, McCarthy, Cohen, & Renshaw, 2010). Next, after this examination of GABA levels across an integrated spectrum of all diseases, we turn to the specific manifestations in individual diagnostic categories.

### Decreased GABA levels in affective disorders

Overall analysis demonstrated no significant differences in GABA levels in individuals with affective disorders compared to HCs; further analysis focusing on MDD revealed lower GABA levels compared to HCs. Our findings support earlier studies indicating lower GABA levels in MDD patients. However, we could not establish a direct relationship between depression severity and GABA levels (Kantrowitz et al., 2021; Song et al., 2021; Wang et al., 2016). Subanalysis based on brain regions found that GABA levels in the OCC decreased in those with affective disorders (Price et al., 2009). Although the OCC has not been accorded a significant role within the neural-circuitry models of affective disorders, positron emission tomography studies have highlighted anomalies in serotonin_1A_ receptor binding and glucose metabolism within this specific region (Saxena et al., 2002). The results indicated that this region merits further consideration.

### Decreased GABA levels in psychosis

No statistically significant differences in GABA levels were found between patients with psychotic disorders and HCs, which contrasts with some previous literature on schizophrenia (Gonzalez-Burgos, Cho, & Lewis, 2015). Our analysis focusing on brain regions nonetheless revealed that individuals with schizophrenia exhibited significantly reduced GABA levels in the ACC. The role of the ACC in cognition, emotion, and self-reference is intricately connected to the abnormalities observed in individuals with schizophrenia (van der Meer, Costafreda, Aleman, & David, 2010). Some researchers have considered the mechanisms through which frontal-cortex circuitry may regulate striatal dopamine, causing dopaminergic dysregulation in psychosis (Howes & Shatalina, 2022). Alterations in GABA receptor subunits could result in altered ligand binding, GABAergic transmission that may negatively contribute to symptoms such as anxiety, and impaired learning and information processing, all of which are disrupted in schizophrenia and BD (Fatemi, Folsom, & Thuras, 2017). Although schizophrenia and BD often share clinical presentations, genetic factors, and neurobiological mechanisms, the results of our study underscore differences in their excitatory amino acid profiles**.**

### Decreased Glu and Glx in affective disorders

Reduced glutamatergic metabolite levels were found in the FC regions of individuals with affective disorders, which may suggest a restricted pool of glutamate-related metabolites, a finding that is consistent with earlier reports showing that glutamate-related metabolites are down-regulated (Yüksel & Öngür, 2010). We should also consider the variability in Glx levels in the FC and further probe its subgroup results. The FC is an important region in affective disorders; it contains the ACC, which is implicated in mood regulation and plays a major role in exhibiting functional abnormalities (Anticevic et al., 2015). The reduced Glu within the major neuronal types could contribute to a degradation of signal integrity in the cortical and hippocampal regions, which is believed to be a neurochemical mechanism underlying the atrophy of neurons in the cortical and limbic brain regions associated with affective disorders (Duman, Sanacora, & Krystal, 2019). Recent studies suggest that impaired glial function may contribute to a reduction in Glu pools in MDD, potentially providing an explanation (Khoodoruth et al., 2022).

### Decreased Glx in neurodevelopmental disorders

Our analysis suggests decreased Glx levels in individuals with neurodevelopmental disorders; this was consistent across the two neurodevelopmental disorders considered here, ASD and ADHD. Extensive and complicated structural and neurochemical processes involving inhibitory and excitatory cortical neurons contribute to the maturation of prefrontal cortical circuitry throughout pre- and postnatal development (Beneyto & Lewis, 2011; Hoftman, Datta, & Lewis, 2017; Snyder & Gao, 2013). Biochemical imbalances may lead to neurodevelopmental disorders. In terms of genetics, Glu transporter genes are functional candidates for autism, and single-nucleotide polymorphisms in the *SLC1A1* and *SLC1A2* Glu transporter genes are associated with autism (Jacob, Landeros-Weisenberger, & Leckman, 2009). Glu receptors are implicated in various cognitive and neuronal developmental processes, which may explain some of the cognitive symptoms (e.g. age-inappropriate inattention) observed in psychiatric patients (Arbanas, 2015; Mahan, 2019).

### Moderators

Our meta-regression found no correlation with age or medication on the alterations of metabolite levels, which is not in line with previous studies (Du et al., 2023). This difference may relate to the disorders or the brain regions considered here. Some ^1^H-MRS studies have begun investigating the potential impact of pharmacological treatments on Glu and Gln concentrations in psychiatric disorders (Evans et al., 2018; Melloni et al., 2022; Merkl et al., 2011). Lower static magnetic-field strengths may lead to spectral overlaps of resonances from different compounds, complicating reliable quantification of ^1^H-MRS-detectable metabolites (Bogner, Hangel, Esmaeili, & Andronesi, 2017); however, we did not find significant results in our meta-regression. This may be due to studies using a 3 T magnetic field being more common while *in vivo* MRS studies on humans using 7 T or higher magnetic fields are still relatively rare, making it difficult to adequately identify the effects of high magnetic fields. Nonetheless, we conducted further subgroup analysis and found that differences in neurotransmitter levels were observed across different magnetic-field strengths. This is possibly attributable to the intrinsic sensitivity of neurotransmitters to magnetic-field intensity. The concentration of GABA, being lower relative to Glu, may exhibit greater fluctuations at lower field strengths; conversely, Glu, with its higher concentration, might require higher magnetic-field strengths for detection of more subtle differences. Moreover, in high magnetic fields, the complexity of spectra and the difficulty of analysis both increase, thereby increasing the need for advanced techniques to correct or compensate for these effects; these then also become potential influencing factors. Adopting a universal sequence can be used to enhance comparability across different edited spectra (Saleh et al., 2019).

### Limitations

The result that many studies have found no significant differences in glutamate levels may be attributed to the dispersed distribution of Glu data across brain regions, along with the selection criteria for the studies included in the research. Research has indicated that patients bearing the greatest disease burden exhibit the most pronounced dysregulation of glutamate mechanisms (Merritt et al., 2023); this dysregulation leads to an increased dependency on pharmacological interventions within these populations. However, the present study excluded research heavily reliant on medication or employing it as an intervention, thus diminishing the observed impact of this factor.

In addition, the finding that most effect sizes were small may also be attributed to heterogeneity and moderating factors. Given this issue, we further analyzed the variability for regions and conducted meta-regression analyses on the moderating variables to examine whether the results were subject to variations due to the influences of other factors. For the CVR results pertaining to the corresponding Hedge's *g*, our interpretation of the outcomes for GABA in the OCC, Glu in the Thal, and Glx in the FC should be approached with caution due to their susceptibility to significant inter-individual variability.

This study also identified significant publication bias concerning certain neurotransmitters in the context of specific mental disorders. This finding underscores the imperative to comprehensively examine all the neurotransmitters associated with mental illnesses and to transcend the confines of categorizing diseases by type, suggesting the necessity to consider mechanisms that span across different disorders.

## Conclusions

The primary results of this ^1^H-MRS meta-analysis indicate reductions in GABA levels across psychiatric disorders, diminished Glx levels in neurodevelopmental disorders, and reductions in Glu levels in the OCC and the PFC in patients with affective disorders. Specific potential moderators did not significantly impact the measurement results of neurotransmitter levels, thereby reinforcing the reliability of our metabolite-level findings. However, further subgroup analyses indicated the importance of considering magnetic-field strength. The reduction in Glu levels was observed more prominently observed in 7T studies due to its higher sensitivity in detecting neurotransmitters. Overall, these results highlight the critical role of GABA and Glu in the etiology of psychiatric disorders and suggest a pattern of GABAergic descent across psychiatric disorders and variations in glutamatergic dysfunction according to specific disorders and brain regions.

## Supporting information

Zhang et al. supplementary material Zhang et al. supplementary material

## Supplementary material

The supplementary material for this article can be found at https://doi.org/10.1017/S0033291724001673
