# Supplementary material for: Charting brain GABA and glutamate levels across psychiatric disorders by quantitative analysis of 121 1H-MRS studies
Source: Psychol Med. 2024 Nov 20;54(15):4071–82. doi: 10.1017/S0033291724001673 (PMC11650199; doi:10.1017/S0033291724001673)
Supplement: Zhang et al. supplementary material [file S0033291724001673sup001.doc]

**Supplementary**

# Charting brain GABA and glutamate levels across psychiatric disorders by quantitative analysis of 121 ^1^H-MRS studies

**Charting the Brain GABA and Glutamate Levels across Psychiatric Disorders by quantitative analysis on 121 ^1^H-MRS Studies**

**Running title:** GABA and Glutamate Levels across Psychiatric Disorders

Zhang Jiayuan^1^, Toulopoulou Timothea^234^, Li Qian^1^, Niu Lijing^1^, Peng Lanxin^1^, Dai Haowei^1^, Chen Keyin^1^, Wang Xingqin, Huang Ruiwang^6^, Wei Xinhua^7*^, Zhang Ruibin^1,8*^

^1^ Laboratory of Cognitive Control and Brain Healthy, Department of Psychology, School of Public Health, Southern Medical University, Guangzhou, PRC China

^2^ Department of Psychology & National Magnetic Resonance Research Center (UMRAM) & Aysel Sabuncu Brain Research Center, Bilkent University, 06800 Ankara, Turkey.

^3^ Department of Psychiatry, National and Kapodistrian University of Athens, Athens, Greece

^4^ Department of Psychiatry, Icahn School of Medicine at Mount Sinai, New York, USA

^5^Department of Neurosurgery, Institute of Brain Diseases, Nanfang Hospital of Southern Medical University, Guangzhou, PRC China

^6^School of Psychology, South China Normal University, Guangzhou, China.

^7^ Department of Radiology, Guangzhou First Affiliated Hospital, Guangzhou, PRC China

^8^Department of Psychiatry, Zhujiang Hospital, Southern Medical University, Guangzhou, PRC China

# Correspondence and request for materials should be addressed to:

Xinhua Wei, Department of Radiology, Guangzhou First Affiliated Hospital, Guangzhou (e-mail: [eyxinhuawei@scut.edu.cn](mailto:eyxinhuawei@scut.edu.cn))

Ruibin Zhang, Department of Psychology, School of Public Health, Southern Medical University (e-mail: [ruibinzhang@foxmail.com](mailto:ruibinzhang@foxmail.com))

**Content**

**Figure S1.** Forest plots of brain GABA levels in affective disorder.

**Figure S2.** Subgroup forest plot of GABA levels (without combining ACC and PFC).

**Figure S3.** Subgroup forest plots of brain GABA levels in affective disorder(A) and neurodevelopmental disorder(B).

**Figure S4.** Forest plots of brain GABA levels in neurodevelopmental disorder.

**Figure S5.** Forest plots of brain GABA levels in psychotic disorder.

**Figure S6.** Forest plots of brain Glu levels in affective disorder.

**Figure S7.** Subgroup forest plot of Glu levels (without combining ACC and PFC).

**Figure S8.** Subgroup forest plots of brain Glu(A) and Glx(B) levels in affective disorder.

**Figure S9.** Subgroup forest plot of Glx levels (without combining ACC and PFC).

**Figure S10.** Forest plots of brain Glx levels in neurodevelopmental disorder.

**Figure S11.** Subgroup forest plots of brain Glu(A) and Glx(B) levels in neurodevelopmental disorder.

**Figure S12.** Subgroup forest plot for field strength on GABA, Glu and Glx levels across all disorders.

**Figure S13.** Forest plot showing the summary effect sizes for the log coefficient of variation ratio (VR) of metabolite measures in patients compared to healthy volunteers (HV).

**Figure S14.** Funnel plots for the studies of GABA on affective disorder, neurodevelopmental disorder, and psychotic disorder.

**Figure S15.** Funnel plots for the studies of Glu on affective disorder, neurodevelopmental disorder, psychotic disorder.

**Figure S16.** Funnel plots for the studies of Glx on affective disorder, neurodevelopmental disorder, and psychotic disorder.

**Table S1.** Clinical and technical characteristics of studies included for the meta-analysis.

**Table S2.** Meta-regression analyses on age, medication status and field strength.

**Included articles in the meta-analyses.**


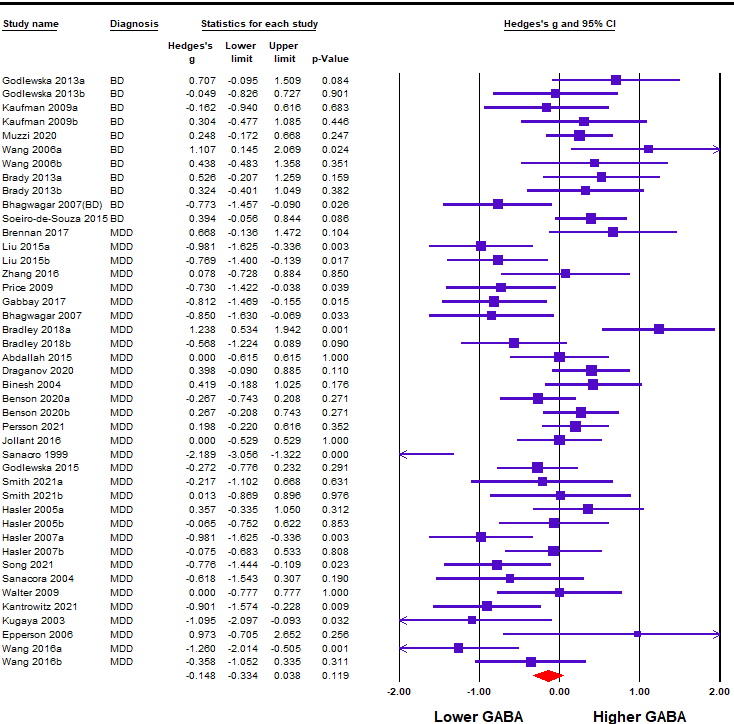


**Figure S1. Forest plots of brain GABA levels in affective disorder.**

The diamond-shaped red symbol represents a summary of effect size. The size of the purple squares is proportionate to the sample size used.


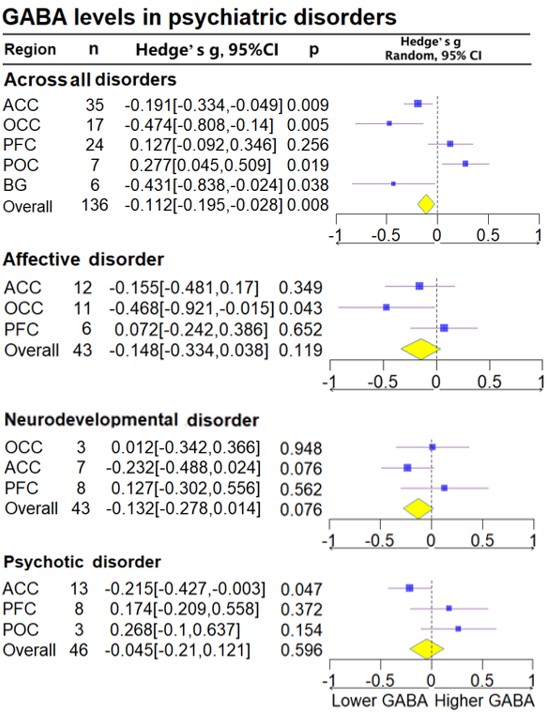


**Figure S2. Subgroup forest plot of GABA levels (without combining ACC and PFC).** ACC, anterior cingulate cortex; PFC, prefrontal cortex; Lower GABA, represent the lower relative deviation from zero as the standard reference point, indicates that the GABA concentration in the patient group is lower than that in the control group when comparing the two populations; Higher GABA, represent the higher relative deviation from zero as the standard reference point. Diamond shaped yellow symbols represent the overall effect. Blue squares represent the subgroup effect and the size is proportionate to the sample size used for each region.


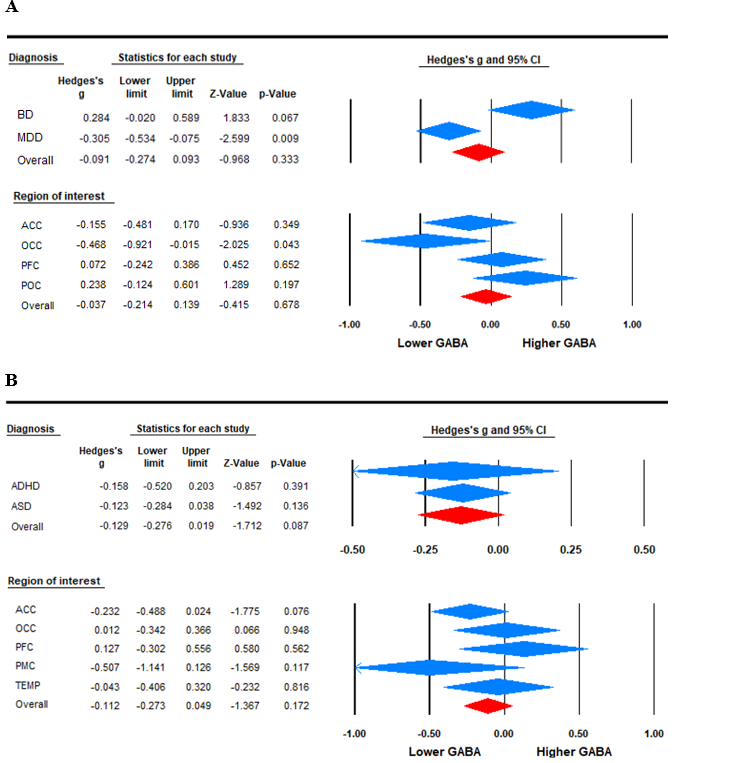


**Figure S3. Subgroup forest plots of brain GABA levels in affective disorder(A) and neurodevelopmental disorder(B).** Diamond shaped red symbols represent the overall effect, and the blue diamond represent the subgroup effect and the size is proportionate to the sample size used for each region.


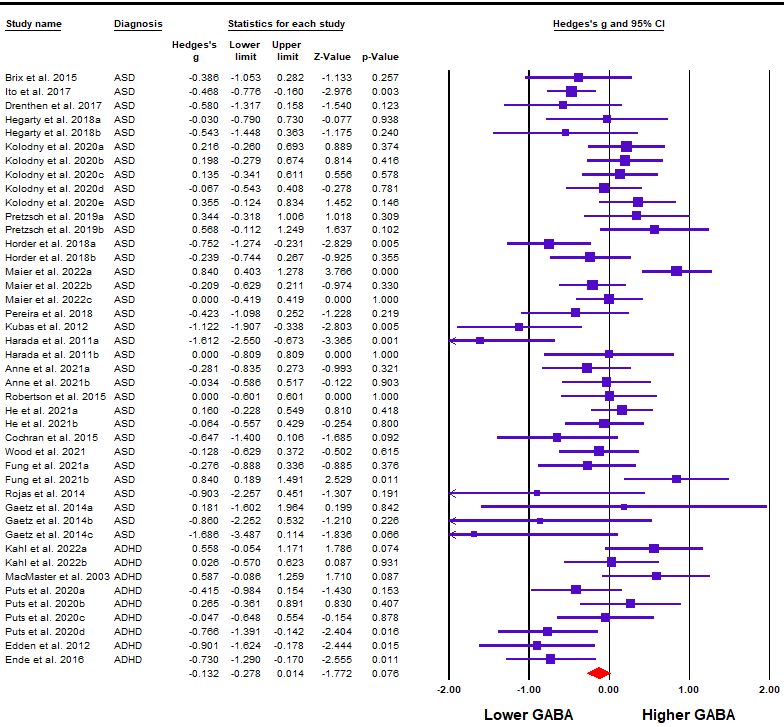


**Figure S4. Forest plots of brain GABA levels in neurodevelopmental disorder.**

The diamond-shaped red symbol represents a summary of effect size. The size of the purple squares is proportionate to the sample size used.


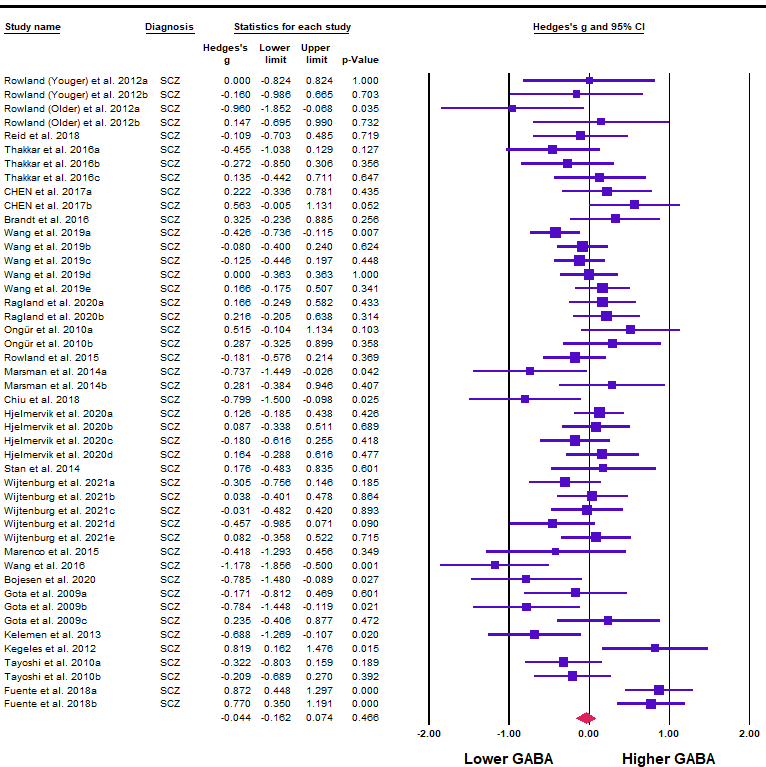


**Figure S5. Forest plots of brain GABA levels in psychotic disorder.**


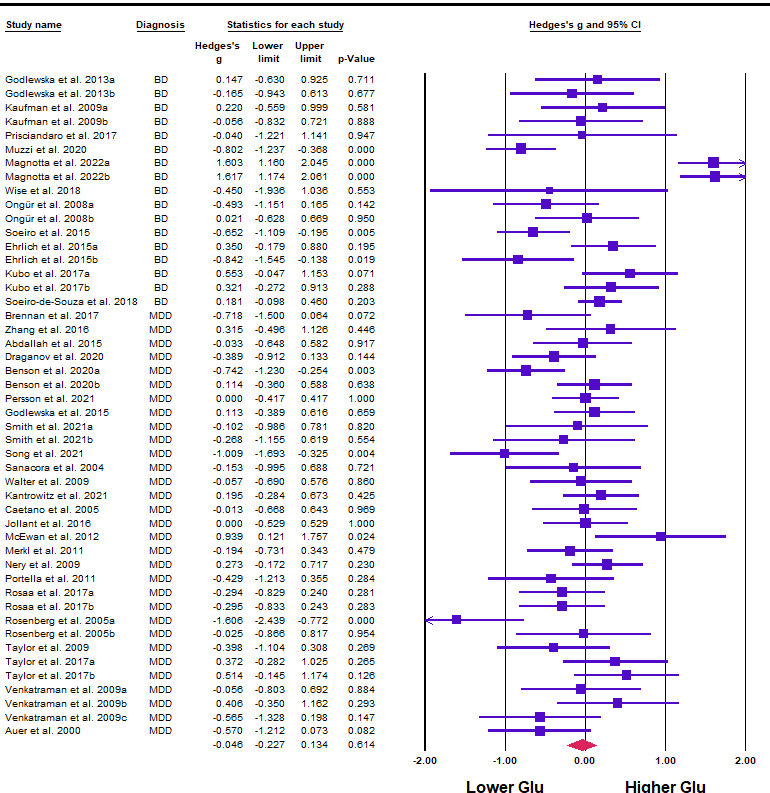


**Figure S6. Forest plots of brain Glu levels in affective disorder.**


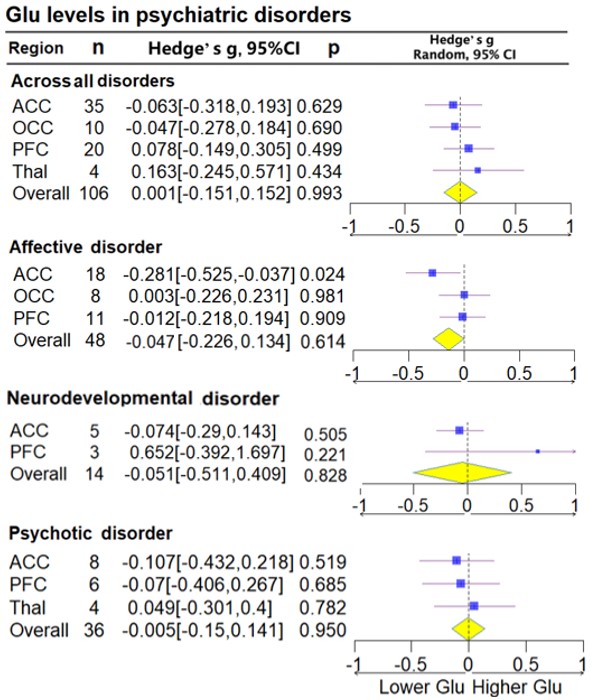


**Figure S7. Subgroup forest plot of brain Glu levels (without combining ACC and PFC)**


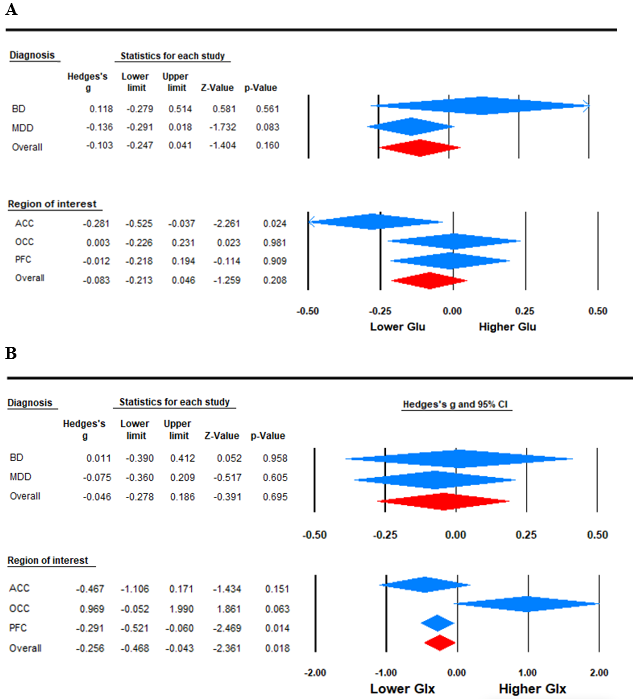


**Figure S8. Subgroup forest plots of brain Glu(A) and Glx(B) levels in affective disorder.**


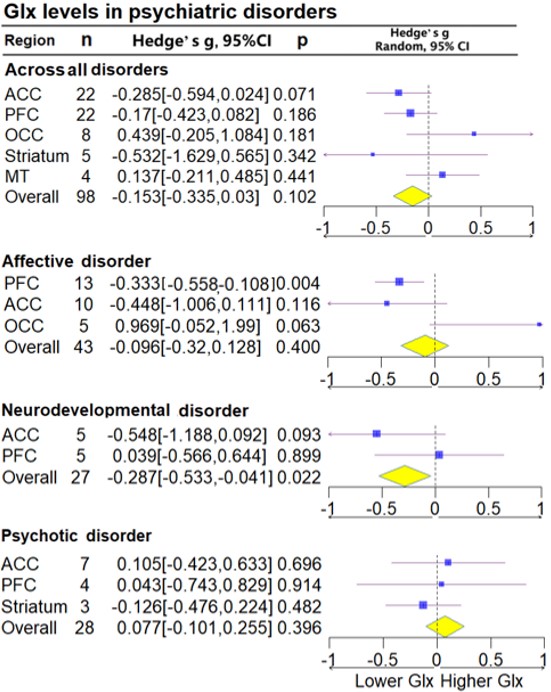


**Figure S9. Subgroup forest plot of brain Glx levels (without combining ACC and PFC)**


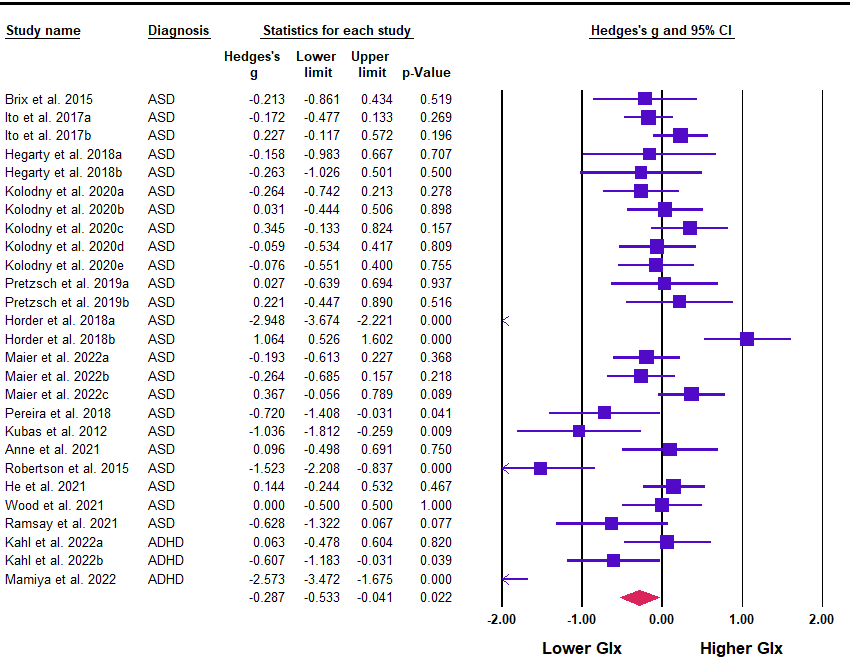


**Figure S10. Forest plots of brain Glx levels in neurodevelopmental disorder.**


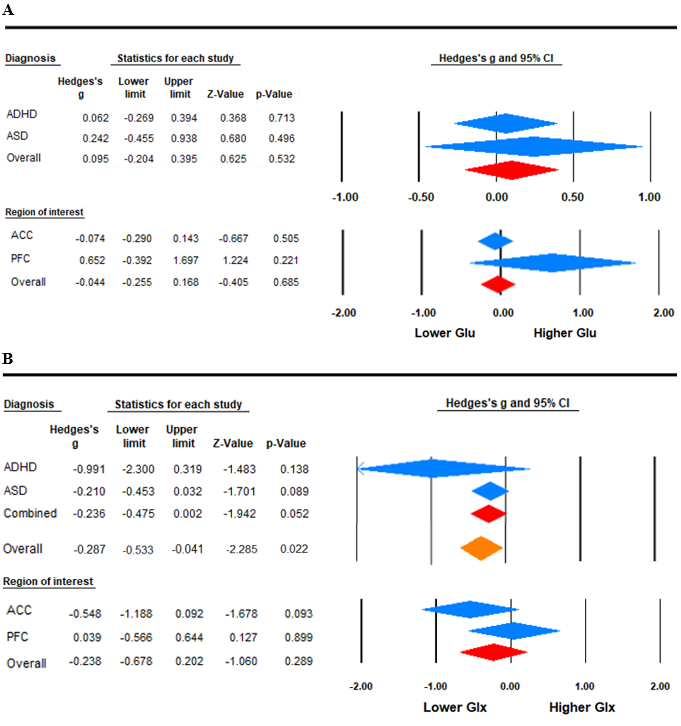


**Figure S11. Subgroup forest plots of brain Glu(A) and Glx(B) levels in neurodevelopmental disorder.**


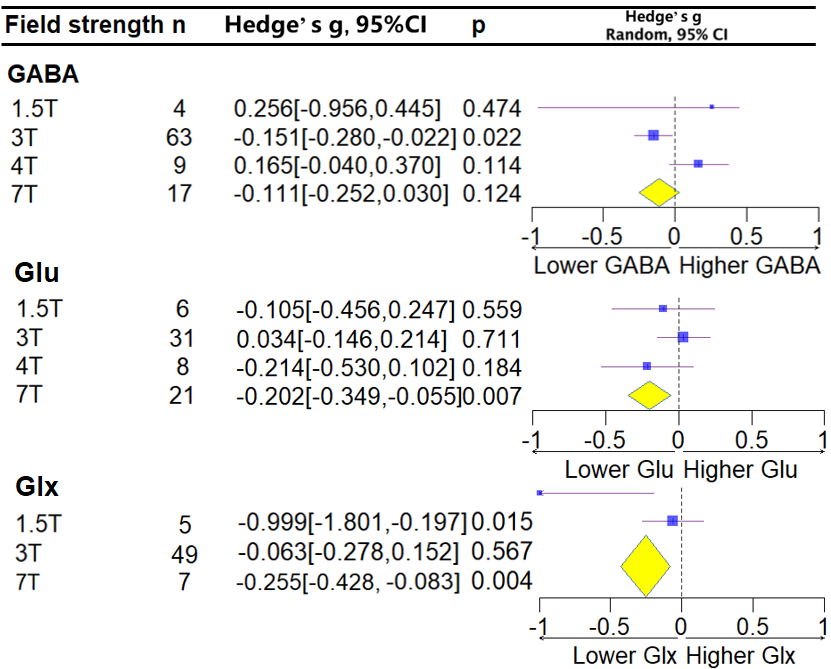
**Figure S12 Subgroup forest plot for field strength on GABA, Glu and Glx levels across all disorders.**


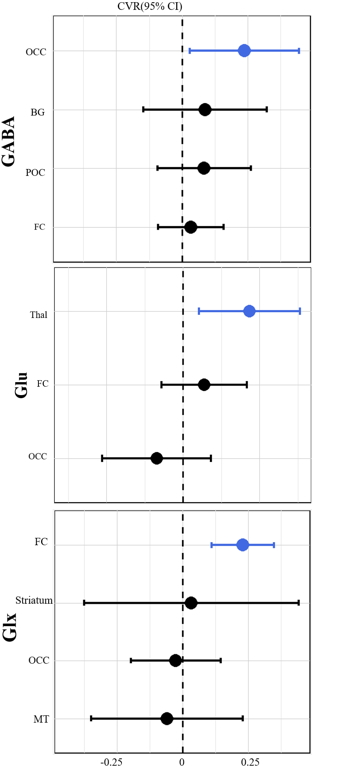


**Figure S13. Forest plot showing the summary effect sizes for the log coefficient of variation ratio (VR) of metabolite measures in patients compared to healthy volunteers (HV).** Significant results are shown in blue. Variability was significantly higher in patients relative to HV in the OCC (all GABAergic metabolites), Thal (all glutamatergic metabolite) and FC (Glx). CVR is defined as mean metabolite levels correlate with standard deviation. CVR, 95% confidence intervals.


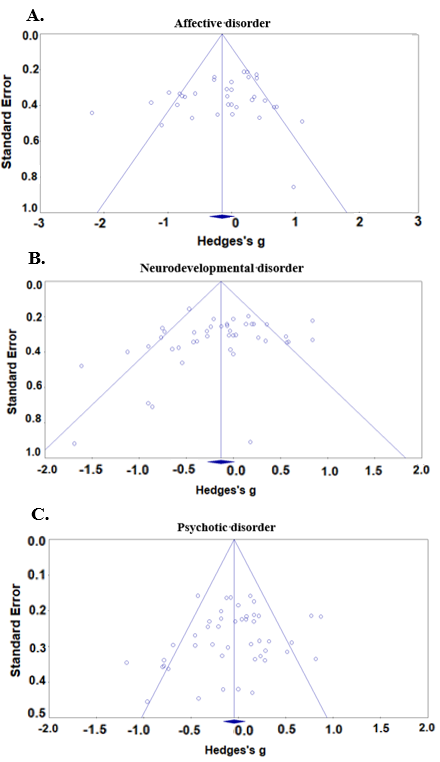


**Figure S14. Funnel plots for the studies of GABA on affective disorder, neurodevelopmental disorder, and psychotic disorder.**


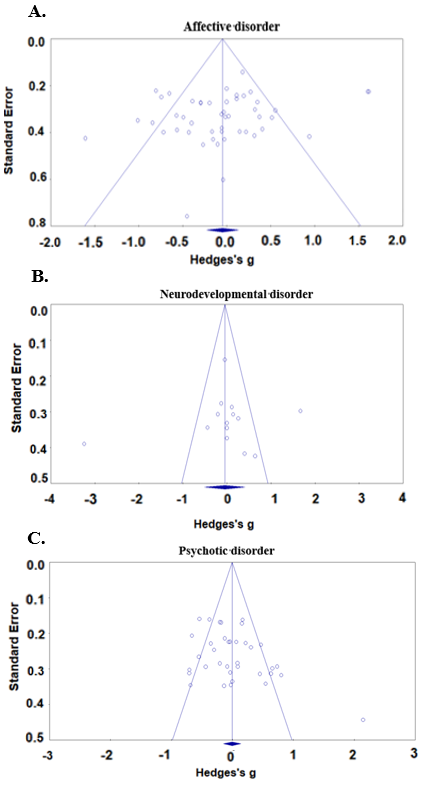


**Figure S15. Funnel plots for the studies of Glu on affective disorder, neurodevelopmental disorder, and psychotic disorder.**


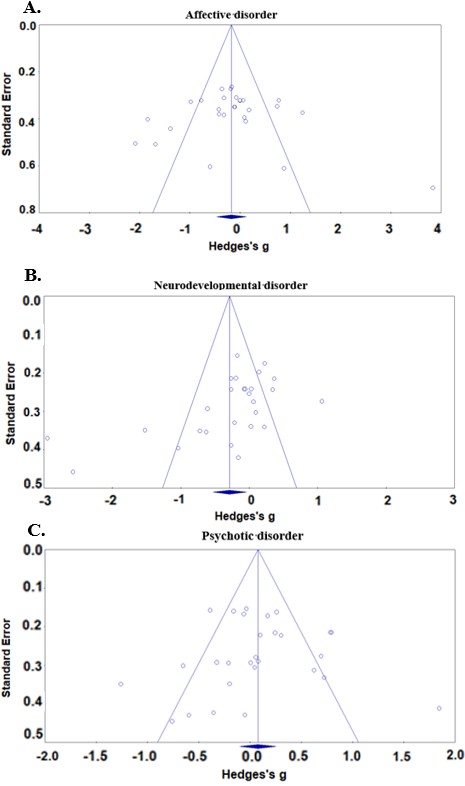


**Figure S16. Funnel plots for the studies of Glx on affective disorder, neurodevelopmental disorder, and psychotic disorder.**

**Table S1. Clinical and technical characteristics of studies included in the meta-analysis.**

| **Study** | **Diagnosis** | | **Measured metabolites** | **Regions of Interest** | **Patient** | | **Control** | | **Male**  **(%)** | **Meds**  **(%, period)** | | **Field**  **strength** | **Editing technique** |
| --- | --- | --- | --- | --- | --- | --- | --- | --- | --- | --- | --- | --- | --- |
|  |  |  |  |  | **N** | **Age (SD)** | **N** | **Age (SD)** |  |  |  |  |  |
| **Affective disorder (61)** | | | | | | | | | | | | | |
| Godlewska et al. 2013 | | BD | GABA/c, Glu/cr | PFC  OCC | 13 | 23.8(3.6) | 11 | 21.9(2.7) | 45.80 | 0 | | 3T | SPECIAL |
| Kaufman et al.2009 | | BD | GABA/Cr, Glu/Cr、Gln/Cr | BG  WB | 13 | 40.5 (12.5) | 11 | 41.2 (14.0) | 37.50 | 100 | | 4T | J-resolved, 2D slab-selective, spin-echo |
| Prisciandaro et al.  2017 | | BD | Glu/Cr | ACC | 20 | 36.3 (11.4) | 19 | 38.0 (11.1) | 44 | 0 | | 3T | SPECIAL |
| Muzzi et al. 2020 | | BD | GABA, Glu, Glu/GABA | ACC | 50 | 33.3(10.6) | 38 | 25.7(5.7) | 47.80 | 100 | | 3T | JPRESS |
| Skok et al. 2016 | | BD | Glx/Cr, Glx/H_2_O | Frontal lobe  TP  ACC | 10 | 43(11.27) | 27 | 40.2(11.99) | 32.40 | 100 | | 1.5 T | PRESS |
| Magnotta et al. 2022 | | BD | Glu, Gln, Glx | Vermis  Putamen | 64 | 39.2(14.0) | 42 | 37.8(13.2) | 35.80 | 100 | | 7T | MP-RAGE,  semi-LASER |
| Wang et al. 2006 | | BD | GABA/Cr | Occipital  mPFC/ACC | 29 | 34.4(12.0) | 12 | 37.2(16.6) | 56.10 | 100 | | 3T | GABA-edited point resolved |
|  | |  |  |  |  |  |  |  |  |  | |  |  |
| Brady et al. 2013 | | BD | GABA/ Cr | ACC  POC | 14 | 32.6(13.6) | 14 | 36.9(10.4) | 64.30% | 100 | | 4T | MEGAPRESS |
| Wise et al. 2018 | | BD | Glu/Cr | ACC | 9 | 31.44(8.23) | 20 | 31.44(8.23) | 20.70% | 100(2 weeks) | | 3T | PRESS |
| Bhagwagar et al. 2007 | | BD | GABA/Cr，Glx/Cr | ACC | 12 | 40.6(4.2) | 11 | 34.3(4.1) | 44.00% | >6months | | 3T | PRESS |
| Öngür et al. 2008 | | BD | Glu, Gln, Gln/Glu | ACC  POC | 15 | 36.3(11.6) | 21 | 34.3(10.0) | 50% | 100 | | 4T | J- PRESS |
| Soeiro-de-Souza  et al. 2015 | | BD | GABA, Glu, Gln, Glu/Gln | ACC | 50 | 31.7(9.1) | 38 | 25.7(5.7) | 47.70 | 100 | | 3T | J- PRESS |
| Ehrlich et al. 2015 | | BD | Glu | ACC  HC | 21 | 45.9(12.3) | 42 | 39.3(7.8) | 61.90 | 95 | | 3T | PRESS |
| Li et al. 2016 | | BD | Glx | ACC, mPFC  PC, PCC | 3 | 31.0(7.6) | 20 | 31.7(11.4) | 48.50 | 100(2 weeks) | | 3T | 2D MRS-PRESS  , PRESS |
| Kubo et al. 2017 | | BD | Glu, Gln, Gln/Glu | ACC  ltBG | 20 | 45.0 | 23 | 46.4 | 65.12 | 100 | | 3T | STEAM |
| Soeiro-de-Souza  et al. 2018 | | BD | Glx/Cr, Glu/Cr | ACC  ltBG | 128 | 32.04 (9.38) | 80 | 28.13 (8.19) | 38.50 | 35.6 (2month) | | 3T | PRESS |
| Brennan et al. 2017 | | MDD | Gln/Glu, Glu/tCr, Gln/tCr, GABA/tCr | ACC | 10 | 38.5 (12.2) | 19 | 38.4 (14.1) | 55.20 | 100(2 weeks) | | 3T | MEGA-PRESS  JPRESS |
| Liu et al. 2015 | | MDD | GABAþ/tCr, Glx/tCr, Glx/GABA+ | ACC  BG | 20 | 23.(1.6) | 20 | 23.6(1.4) | 0 | 100 | | 3T | MEGA-PRESS |
| Zhang et al. 2016 | | MDD | GABA, Glu, Glx | PFC | 11 | 34.09(8.78) | 11 | 33.64(7.187) | 0 | 55 | | 3T | MEGA-PRESS |
| Price et al. 2009 | | MDD | GABA/water | OCC | 24 | 38.3 (12.3) | 18 | 37.25 | 50 | 100（2 weeks） | | 3T | J-edited spin echo |
| Gabbay et al. 2017 | | MDD | GABA/Water, Glx/Water | ACC | 24 | 16.07(2.64) | 15 | 15.33(2.68) | 51.30 | 71 | | 3T | PRESS |
| Bhagwagar et al.  2007 | | MDD | GABA/Cr, Glx/Cr | OCC | 15 | 37.6(14) | 18 | 37.0(13.8) | 48% | 100(3 month） | | 3T | MEGAPRESS |
| Bradley et al. 2018 | | MDD | GABA/Water | Striatal  ACC | 20 | 15.49(2.46) | 16 | 15.56(2.64) | 56% | 100(7 half-lives) | | 3T | J-edited MEGA-PRESS |
| Abdallah et al.2015 | | MDD | GABA, Glu, Gln | OCC | 23 | 43.0(2.2) | 17 | 43.8(3.1) | 72.50% | 100(4 weeks) | | 4T | J-editing |
| Draganov et al.  2020 | | MDD | GABA, Glu, Glx | PFC | 23 | 37.29(10.8) | 54 | 41.77(10.1) | 45.36% | 100(2 weeks) | | 3T | 3D-MPRAGE |
| Benson et al. 2020 | | MDD | GABA+/Cr, Glu/Cr | ACC  POC | 51 | 33.2 (14.4) | 25 | 33.9 (14.6) | 34.20% | 100(2 weeks) (or 5 half-lives) | | 4T | MEGA-PRESS |
| Persson et al. 2021 | | MDD | GABA+/Cr, Glu/Cr, GABA+/Glu | ACC | 42 | 29.2 (9.4) | 45 | 29.5 (11.2) | 44.80% | 100 | | 3T | MEGA-PRESS |
| Bhagwagar et al.  2007 | | MDD | GABA/Cr, Glx/Cr | OCC | 12 | 40.6(4.2) | 11 | 34.3(4.1) | 44.00% | 100(6 months) | | 3T | PRESS |
| Shaw et al. 2019 | | MDD | GABA, Glx | PFC  OCC  Subcortical | 19 | 23 (2.6) | 37 | 21 (1.5) | 0% | 0 | | 3T | MEGA-PRESS |
| Binesh et al. 2004 | | MDD | Glx/Cr, GABA/Cr | DPw | 15 | 72(8) | 33 | 72(8) | 41.67% | 100(several months) | | 1.5T | CHESS |
| Godlewska et al.  2015 | | MDD | GABA, Glu, Gln | OCC | 33 | 29.9(10.6) | 27 | 30.3(10.6) | 41.70% | 100(111  weeks) | | 3T | SPECIAL |
| Smith et al. 2021 | | MDD | GABA/tcr, Glu/tcr | ACC  POC | 9 | 70(7) | 9 | 67(7) | 50% | 100(past year) | | 7T | MP-RAGE |
| Hasler et al. 2005 | | MDD | GABA, Glx | PFC | 16 | 41.0(11.6) | 15 | 27.7(8.8) | 22.60% | 0 | | 7T | (PRESS)-based J-editing |
| Hasler et al. 2007 | | MDD | GABA, Glx | PFC | 20 | 34.0(11.2) | 20 | 34.8(12.4) | 35% | 100(4 weeks) | | 3T | PRESS-based J editing |
| Song et al. 2021 | | MDD | GABA、Glu | MT | 16 | 22.8 (4.1) | 20 | 23.4 (2.1) | 47.37% | N.A. | | 7T | STEAM |
| Sanacora et al.2004 | | MDD | GABA, Glu, Gln | OCC | 19 | 41.9(9.9) | 37 | 35.7(11.4) | 0 | 100(2-weeks) | | 2.1T | J-editing |
| Walter et al. 2009 | | MDD | GABA/Cr, Glu/Cr, Gln/Cr | ACC | 13 | 40.0 | 11 | 34.6 | 31.81% | 100(1 week) | | 3T | single-shot echo-planar |
| Kantrowitz et al.  2021 | | MDD | GABA/Cr, Glu/Cr | P&A | 34 | 37.2(10.7) | 32 | 35.1(9.6) | 53% | 100(14 days) | | 3T | N.A. |
| Kugaya et al. 2003 | | MDD | GABA | OCC | 6 | 36.2(10.4) | 12 | 36.2(10.4) | 100.00% | 100(6 months) | | 2.1T | N.A. |
| Epperson et al. 2002 | | MDD | GABA | OCC | 9 | 30(5.3) | 14 | 31(2.9) | 0.00% | 100(9 months) | | 2.1T | J-editing |
| Block et al. 2009 | | MDD | Glx, Gln/cr | Hippocampus | 31 | 36(10) | 10 | 36(19) | 39% | 100(2 weeks) | | 3T | PRESS |
| Caetano et al. 2005 | | MDD | Glu, Glx | PFC | 14 | 13.3(2.3) | 22 | 13.6(2.8) | 63.89% | 100(7days) | | 1.5T | PRESS |
| Jollant et al. 2016 | | MDD | GABA, Glu | PFC | 24 | 35.0(9.9) | 30 | 35.0(9.9) | 40.74% | 100(7days) | | 3T | SPEACIAL |
| Li et al. 2016 | | MDD | Glx | ACC  PFC  PC  PCC | 33 | 29.9(10.6)  31.0(7.6) | 27 | 30.3(10.6) 31.7(11.4) | 41.70%  48.50% | 100(111weeks) | | 3T | 2D MRS-PRESS, PRESS |
| McEwan et al.2012 | | MDD | Glu/Water | PFC | 12 | 28.67(7.45) | 12 | 29.08(4.89) | 0% | 100(3 months) | | 3T | STEAM |
| Merkl et al. 2011 | | MDD | Glu | PFC | 25 | 51.76(13.16) | 27 | 46.38(12.93) | 26.96% | 0 | | 3T | PRESS |
| MICHAEL et al.  2003 | | MDD | Glx | PFC | 12 | 63.4(10.6) | 12 | 62(8.7) | 41.70% | 100(3~8 days) | | 1.5T | STEAM |
| Milne et al. 2009 | | MDD | Glx | hippocampus | 14 | 32.14 (9.26) | 13 | 30.00 (8.90) | 63% | 7 | | 3T | PRESS |
| Mizrad et al. 2004 | | MDD | Glx | ACC  OCC | 13 | 15.54(2.39) | 13 | 15.36(2.48) | 38.50% | 0 | | 1.5T | PRESS |
| Nery et al. 2009 | | MDD | Glu | ldpc | 37 | 36.6(13.7) | 40 | 40.0(12.3) | 35.10% | 100(2-weeks) | | 1.5T | PRESS |
| Pfleiderer et al.  2003 | | MDD | Glx | ACC | 17 | 61.0(11.2) | 17 | 60.1(10.9) | 29.40% | 100 | | 1.5T | STEAM |
| Portella et al. 2011 | | MDD | Glu, Glx | PFC | 10 | 44.50(8.7) | 15 | 40.47(11.6) | 60% | 100 | | 3T | SVS- PRESS |
| Rosaa et al. 2017 | | MDD | Glu, Glx | PFC  ACC | 31 | 27.7 (4.8) | 23 | 29.0 (6.0) | 0% | N.A. | | 3T | PRESS |
| Rosenberg et al.  2005 | | MDD | Glx, Glu | ACC  OCC | 14 | 15.63(2.33) | 14 | 15.47(2.42) | 35.71% | 0 | | 1.5T | PRESS |
| Taylor et al. 2009 | | MDD | Glx, Glu/cr | ACC | 14 | 32.6 (18–57) | 16 | 31.8 (19–63) | 30% | 79(38 months) | | 3T | PRESS |
| Taylor et al. 2017 | | MDD | Glx, Glu | ACC | 17 | 22.5 (4.6) | 18 | 23.9 (4.6) | 48.60% | 59 | | 7T | STEAM |
| Venkatraman et al.  2009 | | MDD | Glu | PFC  MT  Right | 14 | 72.1(5.3) | 12 | 72.7(4.6) | 46.15% | 100 | | 3T | PRESS |
| Wang et al. 2016 | | MDD | GABA | ACC | 19 | 53.90(2.56) | 13 | 52.62(2.18) | 0.00 | 100(6months) | | 3T | MEGA- PRESS |
| Auer et al. 2000 | | MDD | Glu | ACC | 19 | 50.2(12.2) | 18 | 50.2(12.2) | 37.84 | 100 | | 1.5T | PRESS |
| **Neurodevelopmental disorder (25)** | | | | | | | | | | | | | |
| Kahl et al. 2022 | | ADHD | GABA, Glx | SMA/M1 | 26 | 11.61(2.54) | 25 | 11.12(2.74) | 50% | 0 | | 3T | PRESS |
| Puts et al. 2020 | | ADHD | GABA/Cr, Glu | ACC  DLPFC  PMC  Striatum | 26 | 26(7.70) | 24 | 24(7.38) | 46.00% | 19 | | 7T | STEAM |
| Edden et al.2012 | | ADHD | GABA | PMC | 13 | 10.2 | 19 | 10.6 | 71.90% | 54(1day) | | 3T | J-difference |
| Ende et al. 2016 | | ADHD | GABA, Glu/tCr | Left Frontal | 22 | 30.05(6.7) | 30 | 27.53(6.6) | 0 | 100(2weeks) | | 3T | MEGA-PRESS |
| Bollmann et al. 2015 | | ADHD | GABA+/H_2_O,Glu/H_2_O | ACC | 16 | 38.4 (11.8) | 19 | 31.6 (9.2) | 42.90% | 100(3days) | | 3T | MEGA-PRESS |
| Mamiya et al. 2022 | | ADHD | Glx | ACC | 18 | 42.6 (1.73) | 16 | 41.0 (2.36) | 25% | 22 | | 3T | MPRAGE |
| Brix et al.  2015 | | ASD | GABA+, GABA+/Cr,  Glx | ACC | 14 | 10.2(1.9) | 24 | 10.2(1.8) | 100% | 100 | | 3T | MEGA-PRESS |
| Ito et al. 2017 | | ASD | GABA/Cr, Glu/Cr, Gln/Cr Glx/Cr | ACC  Left Cerebellum | 112(ACC)  114(LC) | 6.4(2.3)  5.6 (2.3) | 65(ACC)  45(LC) | 6.7 (3.1)  6.5 (3.0) | ACC:74.6% LC: 79.9% | 100 | | 3T | Stream, MEGA-PRESS |
| Drenthen et al.2017 | | ASD | Glu/Cr, GABA+/Glu  GABA+/Cr | PFC | 15 | 16.2(1.4) | 18 | 15.3(1.4) | 93.80% | 100(1day) | | 3T | PRESS |
| Hegarty et al. 2018 | | ASD | GABA, Glx | RCH  PFC | 14 | 22.57 (4.36) | 12 | 23.17 (3.04) | 80.80% | 0 | | 3T | MPRAGE, PRESS |
| Kolodny et al. 2020 | | ASD | GABA, Glx | OCC  TEMP  POC | 31 | 22.7 (3.6) | 40 | 23.0 (3.5) | 61.80% | 100 | | 3T | MEGA-PRESS |
| Pretzsch et al. 2019 | | ASD | GABA、Glx | BG  PFC | 17 | 31.29 (9.94) | 17 | 28.47 (6.55) | 100.00% | 30 | | 3T | MEGA-PRESS，IR-FSPGR |
| Horder et al. 2018 | | ASD | GABA, Glu  Gln, Glx | Striatum  PFC | 25 | 30.98 (1.81) | 36 | 28.91(1.40) | N.A. | 100(6 weeks) | | 3T | MEGA-PRESS |
| Maier et al. 2022 | | ASD | GABA、Glx | PFC  ACC | 43 | 34.1(11.7) | 43 | 34.8(10.7) | 73.30% | Y | | 3T | MEGA-PRESS |
| Pereira et al. 2018 | | ASD | GABA, Glx, GABA/tCr, Glx/tCr | PFC | 20 | 13 (2) | 14 | 13 (2) | N.A. | Y | | 3T | MPRAGE |
| Kubas et al. 2012 | | ASD | GABA/Cr, Glx/Cr | BG | 12 | 10.55(4.90) | 16 | 11.35(4.20) | N.A. | 100 | | 1.5T | FLAIR |
| Harada et al. 2011 | | ASD | GABA, Glu | FL  LN | 12 | 5.2 (3.0) | 10 | 5.9(3.2) | N.A. | 100 | | 3T | MEGA-PRESS |
| Anne et al.2021 | | ASD | GABA, Glx, Glx/GABA | OCC  IF | 26 | 32.2 (9.5) | 26 | 30.9 (8.3) | 50 | N.A. | | 3T | MPRAGE |
| Robertson et al.  2015 | | ASD | GABA, Glx, GABA/Glx | VC | 20 | 29.61(9.17) | 21 | 29.10(8.14) | 80.5 | 100 | | 3T | MEGA-PRESS |
| He et al. 2021 | | ASD | GABA, Glx, GABA/Cr, Glx/Cr | SM  Thal | 42 | 10.32(1.49) | 46 | 9.69(1.21) | 79.50 | 100(1 days) | | 3T | MEGA-PRESS |
| Cochran et al. 2015 | | ASD | GABA/Cr, Glu、Gln | ACC | 13 | 14.9(1.5) | 14 | 14.7 (1.8) | 100 | Y | | 3T | PRESS,  MEGA-PRESS |
| Wood et al. 2021 | | ASD | GABA/Cr,  Glx/Cr GABA/Glx | SM | 29 | 14.57(2.7) | 29 | 13.09(3.0) | 67.24 | 37 | | 3T | MEGA‐PRESS |
| Fung et al. 2021 | | ASD | GABA/Water, GABA/Cr + PCr, GABA/Glx, Glx/Cr + PCr | Thal  PFC | 28 | 26.6(8.3) | 29 | 26.6(8.3) | 63.20 | 71 | | 3T | MEGASPECIAL |
| Ramsay et al. 2021 | | ASD | Glx、Glu | ACC | 19 | 37.68 (9.26) | 19 | 38.37 | 100 | 26 | | 3T | MPRAGE |
| Rojas et al. 2014 | | ASD | GABA+/Cr | TEMP | 17 | 14.01 (5.18) | 17 | 12.44 (5.20) | 45.20 | 21 | | 3T | MEGAPRESS |
| Gaetz et al. 2014 | | ASD | GABA | OCC | 17 | 35.3(9.1) | 17 | 35.3(9.1) | 20.59 | 24 | | 3T | MEGA-PRESS |
| **Psychotic disorder** | | | | | | | | | | |  | | |
| Rowland et al.（Youger&Older）2012 | | SZ | GABA. Glx | ACC  CS | 11  10 | 30.2 (6.6) (Young)  51.1 (4.0) (Old) | 10  10 | 33.4 (6.5) (Young)  49.4 (3.9) (Old) | 66.70  70 | 63 | | 3T | MPRAGE |
| Reid et al. 2018 | | SZ | Glu, Gln, GABA, Glu/Gln | ACC | 21 | 23.2 (4.4) | 21 | 23.5 (4.5) | 23.80 | 100 | | 7T | STEAM,  MPRAGE |
| Thakkar et al. 2016 | | SZ | GABA, Glu, Gln, Glx, GABA/Glx, Gln/Glu | OCC  RSTG  LSTG | 21 | 36.4 (7.3) | 24 | 33.9(9.3) | 68.89 | 100 | | 7T | J-difference spectral editing |
| Chen et al. 2017 | | SZ | GABA, Glu, Gln | PFC | 24 | 26.6 (4.7) | 24 | 26.6 (4.7) | 41.67 | 100(2 weeks) | | 3T | MPRAGE |
| Brandt et al. 2016 | | SZ | GABA, Glu, Gln, Glu/Gln | ACC | 27 | 37.5 (16.7) | 27 | 36.6 (14.6) | 72.20 | 100 | | 7T | MPRAGE  STEAM |
| Wang et al. 2019 | | SZ | GABA, Glu, Gln, Glx | ACC/CSO/DLPFC/OFR/Thal | 81 | 22.3 (4.4) | 91 | 23.3 (3.9) | 57.60 | 100 | | 7T | STEAM |
| Ragland et al. 2020 | | SZ | GABA/Cr. Glu/Cr | PFC  ACC | 39 | 23.5(4.6) | 50 | 24.2(4.7) | 68.32 | 77 | | 3T | MEGA-PRESS |
| Öngür et al. 2010 | | SZ | GABA/Cr、Glu/Cr | ACC  POC | 21 | 39 | 19 | 36.3 | 65 | 100 | | 4T | MEGA-PRESS |
| Rowland et al.2015 | | SZ | GABA, Glu, Gln | Frontal lobe | 45 | 37.7 (12.8) | 53 | 37.1 (13.1) | 62.20 | N.A. | | 3T | short-echo |
| Marsman et al. 2014 | | SZ | GABA/Cr, Glu | PC  POC | 13 | 27.6 (6.1) | 19 | 27.7 (5.3) | 27.50 | 100 | | 3T | MEGAsLASER |
| Xin et al. 2016 | | SZ | Glu | PFC | 25 | 24.8 (6.1) | 33 | 25.4(4.5) | 62.10 | 100 | | 3T | SPECIAL |
| Chiu et al. 2018 | | SZ | GABA、Glx | ACC | 19 | 29.11(6.68) | 14 | 27.71(5.88) | 60.60 | 100 | | 3T | MEGA-PRESS |
| Hjelmervik et al. 2020 | | SZ | GABA、Glu、Gln、Glx | LSTG  LIFG  ACC  RSTG | 77 | 29.83(11.48) | 77 | 30.23(10.23) | 100 | 100 | | 3T | PRESS |
| Stan et al. 2014 | | SZ | GABA/cr, Glu/cr | Hippocampus | 18 | 41.94(8.5) | 18 | 35.63(11.74) | 63.9 | 61 | | 3T | (J)difference editing |
| Wijtenburg et al.  2021 | | SZ | GABA, Glu, Gln, Glx, Gln/Glu | ACC  CSO  PFC  Hippocampus  Thal | 39 | 34.2(12.4) | 36 | 30.5(10.5) | 53.85 | 9 | | 7T | MPRAGE |
| Marenco et al. 2015 | | SZ | GABA/cr, GABA/Water | ACC | 25 | 28.4(8.7) | 184 | 30.6(9.2) | 48.80% | 100(14 to 29 days) | | 3T | SPGR |
| Wang et al. 2016 | | SZ | GABA, Glx | PFC | 16 | 22.13 (5.49) | 23 | 22.52(5.50) | 38.70% | 100 | | 3T | 3D-MRPAGE |
| Bojesen et al. 2020 | | SZ | GABA/Cr, Glx/Cr、Glu/Cr | ACC | 11 | 23.0(5.4) | 31 | 22.7(4.4) | N.A. | 100 | | 3T | PRESS |
| Gota et al. 2009 | | SZ | GABA/Cr | Frontal lobe  BG  POC | 18 | 29(11) | 18 | 30(11) | 50% | 100 | | 3T | MEGA-PRESS |
| Kelemen et al.  2013 | | SZ | GABA/Cr | OCC | 28 | 24.9(8.3) | 20 | 24.2(6.9) | 66.70 | 100 | | 3T | N.A. |
| Kegeles et al. 2012 | | SZ | GABA/Water, Glx/Water | PFC | 16 | 32 (11) | 22 | 33(8) | 65.80 | 100(14 days) | | 3T | J-edited |
| Tayoshi et al. 2010 | | SZ | GABA | ACC  BG | 38 | 33.8(9.5) | 29 | 34.9(10.7) | 49.09 | 100 | | 3T | STEAM |
| Fuente-Sandoval et al. 2018 | | SZ | Glx, GABA/Water | PFC | 28 | 23(6.1) | 18 | 23(3.8) | 82.60 | 100 | | 3T | SPGR |
| Fuente-Sandoval et al. 2018 | | SZ | Glx, Glu | DC | 24 | 26.58(8.49) | 18 | 24.56(5.07) | 50% | 100 | | 3T | SPGR |
| Sivaraman et al.  2018 | | SZ | Glx | Striatum | 14 | 22.86 (5.78) | 18 | 23.22 (5.39) | 65.60 | 100 | | 3T | MPRAGE |
| Tarumi et al.  2019 | | SZ | Glx, Glu | ACC  Caudate | 31 | 42.4(12.6) | 29 | 43.7 (11.7) | 45 | 100 | | 3T | PRESS |
| Taylor et al.  2017 | | SZ | Glx, Glu | Thal | 16 | 22.7 (2.9) | 18 | 23.9 (4.6) | 70.60 | 100 | | 7T | STEAM |
| Goldstein et al.  2015 | | SZ | Glu/Cr，Glx/Cr | DPC | 42 | 30.7 (7.2)  35.0 (7.3) | 16 | 33.7 (8.6)  34.1 (7.9) | 56.90 | 0 | | 3T | point resolved spin echo |
| **Other psychiatric disorder** | | | | | | | | | | | | | |
| Prisciandaro et al.  2020 | | AUD | GABA, Glu, Gln | dACC | 23 | 27.00(5.98) | 20 | 24.30(3.16) | 69.80% | 100 | | 3T | 2D J-resolved PRESS |
| Wang et al. 2021 | | AUD | GABA, Glu, Glx | ACC | 23 | 45.70(9.62) | 22 | 46.41(11.67) | 20% | 100(2 weeks) | | 3T | MEGA-PRESS |
| Behar et al. 1999 | | AUD | GABA + homocarnosine, Glu, Gln | OCC | 10 | 35(7) | 5 | 46(11) | N.A. | 0 | | 2.1 T | homonuclear editing |
| Lee et al. 2007 | | AUD | Glu, Glu/Cr | ACC | 13 | 33.8(5.8) | 18 | 32.9(0.9) | N.A. | 100(2 days) | | 1.5 T | GE Signa |
| Thoma et al. 2011 | | AUD | Glu, Gln | Bilateral medial frontal cortex | 7 | 35.50(8.16) | 17 | 32.25(7.85) | 58 | 100 | | 3T | 3-D MPRAGE |
| Hermann et al.2011 | | AUD | Glu | ACC | 47 | 46.3(1.5) | 57 | 45.1(1.5) | 79 | 100(3 months) | | 3 and 9.4 T | FISP |
| Mon et al. 2012 | | AUD | GABA, Glu, Glx | ACC | 20 | 53.9(8.8) | 16 | 49.0(10.1) | 86 | 100 | | 4T | STEAM |

Meds, psychoactive medication use; T, Tesla; Rowland 2013; 2015 are two studies, both distinguishing a young and an old sample. N.A., not available; AUD, alcohol use disorder; ACC, anterior cingulate cortex; BG, basal ganglia; POC, parieto-occipital cortex; PFC, prefrontal cortex; F, frontal cortex; Thal, thalamus cortex; Temp, temporal cortex; OCC, occipital cortex. MPRAGE, Magnetization Prepared Rapid Gradient Echo; SPGR, spoiled gradient echo. FISP, fast imaging with steady precession.

**Table S2. Meta-regression analyses on age, medication status, and field strength.**

|  | **Metabolites** | **Variable** | **Coefficient** | **Standard**  **Error** | **95%**  **Lower** | **95%**  **Upper** | **Z-value** | **2-sided**  ***P*-value** |
| --- | --- | --- | --- | --- | --- | --- | --- | --- |
| **Age** |  |  |  |  |  |  |  |  |
|  | **GABA** | | | | | | | |
|  |  | **Intercept** | -0.215 | 0.141 | -0.492 | 0.062 | -1.52 | 0.128 |
|  |  | **age** | 0.003 | 0.005 | -0.006 | 0.012 | 0.62 | 0.527 |
|  | **Glu** |  |  |  |  |  |  |  |
|  |  | **Intercept** | -0.268 | 0.231 | -0.721 | 0.185 | -1.16 | 0.247 |
|  |  | **age** | 0.008 | 0.007 | -0.005 | 0.021 | 1.21 | 0.226 |
|  |  |  |  |  |  |  |  |  |
|  | **Glx** |  |  |  |  |  |  |  |
|  |  | **Intercept** | 0.049 | 0.258 | -0.456 | 0.555 | 0.19 | 0.848 |
|  |  | **age** | -0.006 | 0.007 | -0.021 | 0.008 | -0.84 | 0.399 |

| **Medication status** |  |  |  |  |  |  |
| --- | --- | --- | --- | --- | --- | --- |

**GABA**

|  | **Intercept** | -0.194 | 0.087 | -0.365 | -0.023 | -2.230 | 0.026 |
| --- | --- | --- | --- | --- | --- | --- | --- |
|  | **Meds** | 0.028 | 0.037 | -0.044 | 0.100 | 0.750 | 0.451 |
| ***Q* = 0.57, *df* = 1** | | | | | | | |
| **Glu** | | | | | | | |
|  | **Intercept** | -0.263 | 0.227 | -0.708 | 0.182 | -1.16 | 0.247 |
|  | **Meds** | 0.008 | 0.007 | -0.005 | 0.021 | 1.21 | 0.226 |
| ***Q* = 1.47*, df* = 1** | | | | | | | |
| **Glx** | | | | | | | |
|  | **Intercept** | 0.01 | 0.159 | -0.302 | 0.322 | 0.06 | 0.95 |
|  | **Meds** | -0.074 | 0.058 | -0.188 | 0.041 | -1.26 | 0.206 |
| ***Q* = 1.60, *df* = 1** | | | | | | | |

**F**ield strength

| **GABA** | | | | | | | |
| --- | --- | --- | --- | --- | --- | --- | --- |
|  | **Intercept** | -0.237 | 0.132 | -0.496 | 0.021 | -1.80 | 0.072 |
|  | **Field strength** | 0.028 | 0.033 | -0.037 | 0.092 | 0.85 | 0.396 |
| ***Q* = 0.72, *df* = 1** | | | | | | | |
| **Glu** | | | | | | | |
|  | **Intercept** | 0.120 | 0.132 | -0.138 | 0.379 | 0.91 | 0.362 |
|  | **Field strength** | -0.044 | 0.028 | -0.099 | 0.011 | -1.58 | 0.114 |
| ***Q* = 2.51, *df* = 1** | | | | | | | |
| **Glx** | | | | | | | |
|  | **Intercept** | -0.241 | 0.246 | -0.723 | 0.240 | -0.98 | 0.325 |
|  | **Field strength** | 0.0262 | 0.067 | -0.105 | 0.157 | 0.39 | 0.696 |
| ***Q*=** **0.15, *df*=1** | | | | | | | |

We encoded the collected medication status data, with the data for metabolite levels divided as follows: receiving treatment, short-term (durations of discontinuation less than six months), long-term (durations of discontinuation more than six months), and never received treatment. We analyzed the collected field strength data for metabolite levels divided as follows: 1.5T; 2.1T; 3T; 4T; 7T. Subsequently, we conducted a meta-regression analysis.

**Included articles in the meta-analyses**

Affective disorder

BD（17）

1．Brady, R. O., McCarthy, J. M., Prescot, A. P., Jensen, J. E., Cooper, A. J., Cohen, B. M., Renshaw, P. F., & Öngür, D. (2013). Brain gamma-aminobutyric acid (GABA) abnormalities in bipolar disorder. Bipolar Disorders, 15(4), 434–439. <https://doi.org/10.1111/bdi.12074>

2．Ehrlich, A., Schubert, F., Pehrs, C., & Gallinat, J. (2015). Alterations of cerebral glutamate in the euthymic state of patients with bipolar disorder. Psychiatry Research: Neuroimaging, 233(2), 73–80. <https://doi.org/10.1016/j.pscychresns.2015.05.010>

3．Galińska-Skok, B., Konarzewska, B., Kubas, B., Tarasów, E., & Szulc, A. (2016). Neurochemical alterations in anterior cingulate cortex in bipolar disorder: A proton magnetic resonance spectroscopy study (1H-MRS). Psychiatria Polska, 50(4), 839–848. https://doi.org/10.12740/PP/58749

4．Godlewska, B. R., Yip, S. W., Near, J., Goodwin, G. M., & Cowen, P. J. (2014). Cortical glutathione levels in young people with bipolar disorder: A pilot study using magnetic resonance spectroscopy. Psychopharmacology, 231(2), 327–332. https://doi.org/10.1007/s00213-013-3244-0

5．Kaufman, R. E., Ostacher, M. J., Marks, E. H., Simon, N. M., Sachs, G. S., Jensen, J. E., Renshaw, P. F., & Pollack, M. H. (2009). Brain GABA levels in patients with bipolar disorder. Progress in Neuro-Psychopharmacology and Biological Psychiatry, 33(3), 427–434. https://doi.org/10.1016/j.pnpbp.2008.12.025

6．Kubo, H., Nakataki, M., Sumitani, S., Iga, J., Numata, S., Kameoka, N., Watanabe, S., Umehara, H., Kinoshita, M., Inoshita, M., Tamaru, M., Ohta, M., Nakayama-Yamauchi, C., Funakoshi, Y., Harada, M., & Ohmori, T. (2017). 1H-magnetic resonance spectroscopy study of glutamate-related abnormality in bipolar disorder. Journal of Affective Disorders, 208, 139–144. https://doi.org/10.1016/j.jad.2016.08.046

7．Li, H., Xu, H., Zhang, Y., Guan, J., Zhang, J., Xu, C., Shen, Z., Xiao, B., Liang, C., Chen, K., Zhang, J., & Wu, R. (2016). Differential neurometabolite alterations in brains of medication-free individuals with bipolar disorder and those with unipolar depression: A two-dimensional proton magnetic resonance spectroscopy study. Bipolar Disorders, 18(7), 583–590. https://doi.org/10.1111/bdi.12445

8．Magnotta, V. A., Xu, J., Fiedorowicz, J. G., Williams, A., Shaffer, J., Christensen, G., Long, J. D., Taylor, E., Sathyaputri, L., Richards, J. G., Harmata, G., & Wemmie, J. (2022). Metabolic abnormalities in the basal ganglia and cerebellum in bipolar disorder: A multi-modal MR study. Journal of Affective Disorders, 301, 390–399. https://doi.org/10.1016/j.jad.2022.01.052

9．Öngür, D., Jensen, J. E., Prescot, A. P., Stork, C., Lundy, M., Cohen, B. M., & Renshaw, P. F. (2008). Abnormal Glutamatergic Neurotransmission and Neuronal-Glial Interactions in Acute Mania. Biological Psychiatry, 64(8), 718–726. https://doi.org/10.1016/j.biopsych.2008.05.014

10．Prisciandaro, J. J., Tolliver, B. K., Prescot, A. P., Brenner, H. M., Renshaw, P. F., Brown, T. R., & Anton, R. F. (2017a). Unique prefrontal GABA and glutamate disturbances in co-occurring bipolar disorder and alcohol dependence. Translational Psychiatry, 7(7), e1163–e1163. https://doi.org/10.1038/tp.2017.141

11．Prisciandaro, J. J., Tolliver, B. K., Prescot, A. P., Brenner, H. M., Renshaw, P. F., Brown, T. R., & Anton, R. F. (2017b). Unique prefrontal GABA and glutamate disturbances in co-occurring bipolar disorder and alcohol dependence. Translational Psychiatry, 7(7), e1163–e1163. https://doi.org/10.1038/tp.2017.141

12．Scotti-Muzzi, E., Chile, T., Moreno, R., Pastorello, B. F., da Costa Leite, C., Henning, A., Otaduy, M. C. G., Vallada, H., & Soeiro-de-Souza, M. G. (2021). ACC Glu/GABA ratio is decreased in euthymic bipolar disorder I patients: possible in vivo neurometabolite explanation for mood stabilization. European archives of psychiatry and clinical neuroscience, 271(3), 537–547. https://doi.org/10.1007/s00406-020-01096-0

13．Soeiro-de-Souza, M. G., Henning, A., Machado-Vieira, R., Moreno, R. A., Pastorello, B. F., da Costa Leite, C., Vallada, H., & Otaduy, M. C. G. (2015). Anterior cingulate Glutamate–Glutamine cycle metabolites are altered in euthymic bipolar I disorder. European Neuropsychopharmacology,25(12),2221–2229. https://doi.org/10.1016/j.euroneuro.2015.0

9.020

14．Soeiro-de-Souza, M. G., Otaduy, M. C. G., Machado-Vieira, R., Moreno, R. A., Nery, F. G., Leite, C., & Lafer, B. (2018). Anterior Cingulate Cortex Glutamatergic Metabolites and Mood Stabilizers in Euthymic Bipolar I Disorder Patients: A Proton Magnetic Resonance Spectroscopy Study. Biological Psychiatry: Cognitive Neuroscience and Neuroimaging, 3(12), 985–991. https://doi.org/10.1016/j.bpsc.2018.02.007

15．Wang, P. W., Sailasuta, N., Chandler, R. A., & Ketter, T. A. (2006). Magnetic resonance spectroscopic measurement of cerebral gamma-aminobutyric acid concentrations in patients with bipolar disorders. Acta Neuropsychiatrica, 18(2), 120–126. https://doi.org/10.1111/j.1601-5215.2006.00132.x

16．Wise, T., Taylor, M. J., Herane-Vives, A., Gammazza, A. M., Cappello, F., Lythgoe, D. J., Williams, S. C., Young, A. H., Cleare, A. J., & Arnone, D. (2018). Glutamatergic hypofunction in medication-free major depression: Secondary effects of affective diagnosis and relationship to peripheral glutaminase. Journal of Affective Disorders, 234, 214–219. https://doi.org/10.1016/j.jad.2018.02.059

17．Xu, J., Dydak, U., Harezlak, J., Nixon, J., Dzemidzic, M., Gunn, A. D., Karne, H. S., & Anand, A. (2013). Neurochemical abnormalities in unmedicated bipolar depression and mania: A 2D 1H MRS investigation. Psychiatry Research: Neuroimaging, 213(3), 235–241. https://doi.org/10.1016/j.pscychresns.2013.02.008

MDD（44）

1. Abdallah, C. G., Jiang, L., De Feyter, H. M., Fasula, M., Krystal, J. H., Rothman, D. L., Mason, G. F., & Sanacora, G. (2014). Glutamate Metabolism in Major Depressive Disorder. American Journal of Psychiatry, 171(12), 1320–1327. https://doi.org/10.1176/appi.ajp.2014.14010067
2. Auer, D. P., Pütz, B., Kraft, E., Lipinski, B., Schill, J., & Holsboer, F. (2000). Reduced glutamate in the anterior cingulate cortex in depression: an in vivo proton magnetic resonance spectroscopy study. Biological psychiatry, 47(4), 305–313. https://doi.org/10.1016/s0006-3223(99)00159-6
3. Benson, K. L., Bottary, R., Schoerning, L., Baer, L., Gonenc, A., Eric Jensen, J., & Winkelman, J. W. (2020). 1H MRS Measurement of Cortical GABA and Glutamate in Primary Insomnia and Major Depressive Disorder: Relationship to Sleep Quality and Depression Severity. Journal of Affective Disorders, 274, 624–631. https://doi.org/10.1016/j.jad.2020.05.026
4. Bhagwagar, Z., Wylezinska, M., Jezzard, P., Evans, J., Ashworth, F., Sule, A., Matthews, P. M., & Cowen, P. J. (2007). Reduction in Occipital Cortex γ-Aminobutyric Acid Concentrations in Medication-Free Recovered Unipolar Depressed and Bipolar Subjects. Biological Psychiatry, 61(6), 806–812. https://doi.org/10.1016/j.biopsych.2006.08.048

5．Bhagwagar, Z., Wylezinska, M., Jezzard, P., Evans, J., Boorman, E., M. Matthews, P., & J. Cowen, P. (2008). Low GABA concentrations in occipital cortex and anterior cingulate cortex in medication-free, recovered depressed patients. The International Journal of Neuropsychopharmacology, 11(02). https://doi.org/10.1017/S1461145707007924

6．Binesh, N., Kumar, A., Hwang, S., Mintz, J., & Thomas, M. A. (2004). Neurochemistry of late-life major depression: A pilot two-dimensional MR spectroscopic study. Journal of Magnetic Resonance Imaging, 20(6), 1039–1045. https://doi.org/10.1002/jmri.20214

7．Block, W., Träber, F., von Widdern, O., Metten, M., Schild, H., Maier, W., Zobel, A., & Jessen, F. (2009). Proton MR spectroscopy of the hippocampus at 3 T in patients with unipolar major depressive disorder: Correlates and predictors of treatment response. The International Journal of Neuropsychopharmacology, 12(03), 415. https://doi.org/10.1017/S1461145708009516

8．Bradley, K. A., Alonso, C. M., Mehra, L. M., Xu, J., & Gabbay, V. (2018). Elevated striatal γ-aminobutyric acid in youth with major depressive disorder. Progress in Neuro-Psychopharmacology and Biological Psychiatry, 86, 203–210. https://doi.org/10.1016/j.pnpbp.2018.06.004

9．Brennan, B. P., Admon, R., Perriello, C., LaFlamme, E. M., Athey, A. J., Pizzagalli, D. A., Hudson, J. I., Pope, H. G., & Jensen, J. E. (2017). Acute change in anterior cingulate cortex GABA, but not glutamine/glutamate, mediates antidepressant response to citalopram. Psychiatry Research: Neuroimaging, 269, 9–16. https://doi.org/10.1016/j.pscychresns.2017.08.009

10．Caetano, S. C., Fonseca, M., Olvera, R. L., Nicoletti, M., Hatch, J. P., Stanley, J. A., Hunter, K., Lafer, B., Pliszka, S. R., & Soares, J. C. (2005). Proton spectroscopy study of the left dorsolateral prefrontal cortex in pediatric depressed patients. Neuroscience Letters, 384(3), 321–326. https://doi.org/10.1016/j.neulet.2005.04.099

11．Colic, L., von Düring, F., Denzel, D., Demenescu, L. R., Lord, A. R., Martens, L., Lison, S., Frommer, J., Vogel, M., Kaufmann, J., Speck, O., Li, M., & Walter, M. (2019). Rostral Anterior Cingulate Glutamine/Glutamate Disbalance in Major Depressive Disorder Depends on Symptom Severity. Biological Psychiatry: Cognitive Neuroscience and Neuroimaging, 4(12), 1049–1058. https://doi.org/10.1016/j.bpsc.2019.04.003

12．Draganov, M., Vives-Gilabert, Y., de Diego-Adeliño, J., Vicent-Gil, M., Puigdemont, D., & Portella, M. J. (2020). Glutamatergic and GABA-ergic abnormalities in First-episode depression. A 1-year follow-up 1H-MR spectroscopic study. Journal of Affective Disorders, 266, 572–577. https://doi.org/10.1016/j.jad.2020.01.138

13．Epperson, C. N., Gueorguieva, R., Czarkowski, K. A., Stiklus, S., Sellers, E., Krystal, J. H., Rothman, D. L., & Mason, G. F. (2006). Preliminary evidence of reduced occipital GABA concentrations in puerperal women: A 1H-MRS study. Psychopharmacology, 186(3), 425–433. https://doi.org/10.1007/s00213-006-0313-7

14．Gabbay, V., Bradley, K. A., Mao, X., Ostrover, R., Kang, G., & Shungu, D. C. (2017). Anterior cingulate cortex γ-aminobutyric acid deficits in youth with depression. Translational Psychiatry, 7(8), e1216–e1216. https://doi.org/10.1038/tp.2017.187

15．Godlewska, B. R., Near, J., & Cowen, P. J. (2015). Neurochemistry of major depression: A study using magnetic resonance spectroscopy. Psychopharmacology, 232(3), 501–507. https://doi.org/10.1007/s00213-014-3687-y

16．Hasler, G., Meyers, N., Shen, J., & Drevets, W. C. (2007). Reduced Prefrontal Glutamate/Glutamine and ␥-Aminobutyric Acid Levels in Major Depression Determined Using Proton Magnetic Resonance Spectroscopy. ARCH GEN PSYCHIATRY, 64.

17．Hasler, G., Neumeister, A., van der Veen, J. W., Tumonis, T., Bain, E. E., Shen, J., Drevets, W. C., & Charney, D. S. (2005). Normal Prefrontal Gamma-Aminobutyric Acid Levels in Remitted Depressed Subjects Determined by Proton Magnetic Resonance Spectroscopy. Biological Psychiatry, 58(12), 969–973. https://doi.org/10.1016/j.biopsych.2005.05.017

18．Jollant, F., Richard-Devantoy, S., Ding, Y., Turecki, G., Bechara, A., & Near, J. (2016). Prefrontal inositol levels and implicit decision-making in healthy individuals and depressed patients. European Neuropsychopharmacology, 26(8), 1255–1263. https://doi.org/10.1016/j.euroneuro.2016.06.005

19．Kantrowitz, J. T., Dong, Z., Milak, M. S., Rashid, R., Kegeles, L. S., Javitt, D. C., Lieberman, J. A., & John Mann, J. (2021). Ventromedial prefrontal cortex/anterior cingulate cortex Glx, glutamate, and GABA levels in medication-free major depressive disorder. Translational Psychiatry, 11(1), 419. https://doi.org/10.1038/s41398-021-01541-1

20．Kugaya, A., Sanacora, G., Verhoeff, N. P. L. G., Fujita, M., Mason, G. F., Seneca, N. M., Bozkurt, A., Khan, S. A., Anand, A., Degen, K., Charney, D. S., Zoghbi, S. S., Baldwin, R. M., Seibyl, J. P., & Innis, R. B. (2003). Cerebral benzodiazepine receptors in depressed patients measured with [123i]iomazenil SPECT. Biological Psychiatry, 54(8), 792–799. https://doi.org/10.1016/S0006-3223(02)01788-2

21．Liu, B., Wang, G., Gao, D., Gao, F., Zhao, B., Qiao, M., Yang, H., Yu, Y., Ren, F., Yang, P., Chen, W., & Rae, C. D. (2015). Alterations of GABA and glutamate–glutamine levels in premenstrual dysphoric disorder: A 3T proton magnetic resonance spectroscopy study. Psychiatry Research: Neuroimaging, 231(1), 64–70. https://doi.org/10.1016/j.pscychresns.2014.10.020

22．McEwen, A. M., Burgess, D. T. A., Hanstock, C. C., Seres, P., Khalili, P., Newman, S. C., Baker, G. B., Mitchell, N. D., Khudabux-Der, J., Allen, P. S., & LeMelledo, J.-M. (2012). Increased Glutamate Levels in the Medial Prefrontal Cortex in Patients with Postpartum Depression. Neuropsychopharmacology, 37(11), 2428–2435. https://doi.org/10.1038/npp.2012.101

23．Merkl, A., Schubert, F., Quante, A., Luborzewski, A., Brakemeier, E.-L., Grimm, S., Heuser, I., & Bajbouj, M. (2011). Abnormal Cingulate and Prefrontal Cortical Neurochemistry in Major Depression After Electroconvulsive Therapy. Biological Psychiatry, 69(8), 772–779. https://doi.org/10.1016/j.biopsych.2010.08.009

24．Michael, N., Erfurth, A., Ohrmann, P., Arolt, V., Heindel, W., & Pfleiderer, B. (2003). Metabolic changes within the left dorsolateral prefrontal cortex occurring with electroconvulsive therapy in patients with treatment resistant unipolar depression. Psychological Medicine, 33(7), 1277–1284. https://doi.org/10.1017/S0033291703007931

25．Milne, A., MacQueen, G. M., Yucel, K., Soreni, N., & Hall, G. B. C. (2009). Hippocampal metabolic abnormalities at first onset and with recurrent episodes of a major depressive disorder: A proton magnetic resonance spectroscopy study. NeuroImage, 47(1), 36–41. https://doi.org/10.1016/j.neuroimage.2009.03.031

26．Mirza, Y., Tang, J., Russell, A., Banerjee, S. P., Bhandari, R., Ivey, J., Rose, M., Moore, G. J., & Rosenberg, D. R. (2004). Reduced Anterior Cingulate Cortex Glutamatergic Concentrations in Childhood Major Depression. Journal of the American Academy of Child & Adolescent Psychiatry, 43(3), 341–348. https://doi.org/10.1097/00004583-200403000-00017

27．Nery, F. G., Stanley, J. A., Chen, H.-H., Hatch, J. P., Nicoletti, M. A., Monkul, E. S., Matsuo, K., Caetano, S. C., Peluso, M. A., Najt, P., & Soares, J. C. (2009). Normal metabolite levels in the left dorsolateral prefrontal cortex of unmedicated major depressive disorder patients: A single voxel 1H spectroscopy study. Psychiatry Research: Neuroimaging, 174(3), 177–183. https://doi.org/10.1016/j.pscychresns.2009.05.003

28．Persson, J., Wall, A., Weis, J., Gingnell, M., Antoni, G., Lubberink, M., & Bodén, R. (2021). Inhibitory and excitatory neurotransmitter systems in depressed and healthy: A positron emission tomography and magnetic resonance spectroscopy study. Psychiatry Research: Neuroimaging, 315, 111327. https://doi.org/10.1016/j.pscychresns.2021.111327

29．Pfleiderer, B., Michael, N., Erfurth, A., Ohrmann, P., Hohmann, U., Wolgast, M., Fiebich, M., Arolt, V., & Heindel, W. (2003). Effective electroconvulsive therapy reverses glutamate/glutamine deficit in the left anterior cingulum of unipolar depressed patients. Psychiatry Research: Neuroimaging, 122(3), 185–192. https://doi.org/10.1016/S0925-4927(03)00003-9

30．Portella, M. J., de Diego-Adeliño, J., Gómez-Ansón, B., Morgan-Ferrando, R., Vives, Y., Puigdemont, D., Pérez-Egea, R., Ruscalleda, J., Enric Álvarez, & Pérez, V. (2011). Ventromedial prefrontal spectroscopic abnormalities over the course of depression: A comparison among first episode, remitted recurrent and chronic patients. Journal of Psychiatric Research, 45(4), 427–434. https://doi.org/10.1016/j.jpsychires.2010.08.010

31．Price, R. B., Shungu, D. C., Mao, X., Nestadt, P., Kelly, C., Collins, K. A., Murrough, J. W., Charney, D. S., & Mathew, S. J. (2009). Amino Acid Neurotransmitters Assessed by Proton Magnetic Resonance Spectroscopy: Relationship to Treatment Resistance in Major Depressive Disorder. Biological Psychiatry, 65(9), 792–800. https://doi.org/10.1016/j.biopsych.2008.10.025

32．Rosa, C. E., Soares, J. C., Figueiredo, F. P., Cavalli, R. C., Barbieri, M. A., Schaufelberger, M. S., Salmon, C. E. G., Del-Ben, C. M., & Santos, A. C. (2017). Glutamatergic and neural dysfunction in postpartum depression using magnetic resonance spectroscopy. Psychiatry Research: Neuroimaging, 265, 18–25. https://doi.org/10.1016/j.pscychresns.2017.04.008

33．Rosenberg, D. R., MacMaster, F. P., Mirza, Y., Smith, J. M., Easter, P. C., Banerjee, S. P., Bhandari, R., Boyd, C., Lynch, M., Rose, M., Ivey, J., Villafuerte, R. A., Moore, G. J., & Renshaw, P. (2005). Reduced Anterior Cingulate Glutamate in Pediatric Major Depression: A Magnetic Resonance Spectroscopy Study. Biological Psychiatry, 58(9), 700–704. https://doi.org/10.1016/j.biopsych.2005.05.007

34．Sanacora, G., Gueorguieva, R., Epperson, C. N., Wu, Y.-T., Appel, M., Rothman, D. L., Krystal, J. H., & Mason, G. F. (2004). Subtype-Specific Alterations of ␥-Aminobutyric Acid and Glutamate in Patients With Major Depression. ARCH GEN PSYCHIATRY, 61.

35．Sanacora, G., Mason, G. F., Rothman, D. L., Behar, K. L., Hyder, F., Petroff, O. A. C., Berman, R. M., Charney, D. S., & Krystal, J. H. (1999). Reduced Cortical ³-Aminobutyric Acid Levels in Depressed Patients Determined by Proton Magnetic Resonance Spectroscopy. ARCH GEN PSYCHIATRY, 56.

36．Shaw, A., Brealy, J., Richardson, H., Muthukumaraswamy, S. D., Edden, R. A., John Evans, C., Puts, N. A. J., Singh, K. D., & Keedwell, P. A. (2013). Marked Reductions in Visual Evoked Responses But Not γ-Aminobutyric Acid Concentrations or γ-Band Measures in Remitted Depression. Biological Psychiatry, 73(7), 691–698. https://doi.org/10.1016/j.biopsych.2012.09.032

37．Smith, G. S., Oeltzschner, G., Gould, N. F., Leoutsakos, J.-M. S., Nassery, N., Joo, J. H., Kraut, M. A., Edden, R. A. E., Barker, P. B., Wijtenburg, S. A., Rowland, L. M., & Workman, C. I. (2021). Neurotransmitters and Neurometabolites in Late-Life Depression: A Preliminary Magnetic Resonance Spectroscopy Study at 7T. Journal of Affective Disorders, 279, 417–425. https://doi.org/10.1016/j.jad.2020.10.011

38．Song, X. M., Hu, X.-W., Li, Z., Gao, Y., Ju, X., Liu, D.-Y., Wang, Q.-N., Xue, C., Cai, Y.-C., Bai, R., Tan, Z.-L., & Northoff, G. (2021). Reduction of higher-order occipital GABA and impaired visual perception in acute major depressive disorder. Molecular Psychiatry, 26(11), 6747–6755. https://doi.org/10.1038/s41380-021-01090-5

39．Taylor, M. J., Selvaraj, S., Norbury, R., Jezzard, P., & Cowen, P. J. (2009). Normal glutamate but elevated myo-inositol in anterior cingulate cortex in recovered depressed patients. Journal of Affective Disorders, 119(1–3), 186–189. https://doi.org/10.1016/j.jad.2009.02.022

40．Taylor, R., Osuch, E. A., Schaefer, B., Rajakumar, N., Neufeld, R. W. J., Théberge, J., & Williamson, P. C. (2017). Neurometabolic abnormalities in schizophrenia and depression observed with magnetic resonance spectroscopy at 7 T. BJPsych Open, 3(1), 6–11. https://doi.org/10.1192/bjpo.bp.116.003756

41．Venkatraman, T. N., Krishnan, R. R., Steffens, D. C., Song, A. W., & Taylor, W. D. (2009). Biochemical abnormalities of the medial temporal lobe and medial prefrontal cortex in late-life depression. Psychiatry Research: Neuroimaging, 172(1), 49–54. https://doi.org/10.1016/j.pscychresns.2008.07.001

42．Walter, M., Henning, A., Grimm, S., Schulte, R. F., Beck, J., Dydak, U., Schnepf, B., Boeker, H., Boesiger, P., & Northoff, G. (2009). The Relationship Between Aberrant Neuronal Activation in the Pregenual Anterior Cingulate, Altered Glutamatergic Metabolism, and Anhedonia in Major Depression. Archives of General Psychiatry, 66(5), 478. https://doi.org/10.1001/archgenpsychiatry.2009.39

43．Wang, Z., Zhang, A., Zhao, B., Gan, J., Wang, G., Gao, F., Liu, B., Gong, T., Liu, W., & Edden, R. A. E. (2016). GABA+ levels in postmenopausal women with mild-to-moderate depression: A preliminary study. Medicine, 95(39), e4918. https://doi.org/10.1097/MD.0000000000004918

44．Zhang, X., Tang, Y., Maletic-Savatic, M., Sheng, J., Zhang, X., Zhu, Y., Zhang, T., Wang, J., Tong, S., Wang, J., & Li, Y. (2016). Altered neuronal spontaneous activity correlates with glutamate concentration in medial prefrontal cortex of major depressed females: An fMRI-MRS study. Journal of Affective Disorders, 201, 153–161. https://doi.org/10.1016/j.jad.2016.05.014

Developmental disorder

ADHD（6）

１．Bollmann, S., Ghisleni, C., Poil, S.-S., Martin, E., Ball, J., Eich-Höchli, D., Edden, R. A. E., Klaver, P., Michels, L., Brandeis, D., & O’Gorman, R. L. (2015). Developmental changes in gamma-aminobutyric acid levels in attention-deficit/hyperactivity disorder. Translational Psychiatry, 5(6), e589–e589. https://doi.org/10.1038/tp.2015.79

２．Edden, R. A., Crocetti, D., Zhu, H., Gilbert, D. L., & Mostofsky, S. H. (2012). Reduced GABA concentration in attention-deficit/hyperactivity disorder. Archives of general psychiatry, 69(7), 750–753. https://doi.org/10.1001/archgenpsychiatry.2011.2280

3．Ende, G., Cackowski, S., Van Eijk, J., Sack, M., Demirakca, T., Kleindienst, N., Bohus, M., Sobanski, E., Krause-Utz, A., & Schmahl, C. (2016). Impulsivity and Aggression in Female BPD and ADHD Patients: Association with ACC Glutamate and GABA Concentrations. Neuropsychopharmacology : official publication of the American College of Neuropsychopharmacology, 41(2), 410–418. https://doi.org/10.1038/npp.2015.153

4．Kahl, C., Swansburg, R., Hai, T., Wrightson, J., Bell, T., Lemay, J.-F., Kirton, A., & MacMaster, F. (2021). Differences in Neurometabolites and Transcranial Magnetic Stimulation Motor Maps in Children With Attention-Deficit Hyperactivity Disorder. Biological Psychiatry, 89(9), S193–S194. https://doi.org/10.1016/j.biopsych.2021.02.491

5．Mamiya, P. C., Richards, T. L., Edden, R. A. E., Lee, A. K. C., Stein, M. A., & Kuhl, P. K. (2022). Reduced Glx and GABA Inductions in the Anterior Cingulate Cortex and Caudate Nucleus Are Related to Impaired Control of Attention in Attention-Deficit/Hyperactivity Disorder. International Journal of Molecular Sciences, 23(9), 4677. https://doi.org/10.3390/ijms23094677

6．Puts, N. A., Ryan, M., Oeltzschner, G., Horska, A., Edden, R. A. E., & Mahone, E. M. (2020). Reduced striatal GABA in unmedicated children with ADHD at 7T. Psychiatry Research: Neuroimaging, 301, 111082. https://doi.org/10.1016/j.pscychresns.2020.111082

ASD（１9）

１．Brix, M. K., Ersland, L., Hugdahl, K., Grüner, R., Posserud, M.-B., Hammar, Å., Craven, A. R., Noeske, R., Evans, C. J., Walker, H. B., Midtvedt, T., & Beyer, M. K. (2015). “Brain MR spectroscopy in autism spectrum disorder—The GABA excitatory/inhibitory imbalance theory revisited”. Frontiers in Human Neuroscience, 9. https://doi.org/10.3389/fnhum.2015.00365

2．Carvalho Pereira, A., Violante, I. R., Mouga, S., Oliveira, G., & Castelo-Branco, M. (2018). Medial Frontal Lobe Neurochemistry in Autism Spectrum Disorder is Marked by Reduced N-Acetylaspartate and Unchanged Gamma-Aminobutyric Acid and Glutamate + Glutamine Levels. Journal of autism and developmental disorders, 48(5), 1467–1482. https://doi.org/10.1007/s10803-017-3406-8

3．Cochran, D. M., Sikoglu, E. M., Hodge, S. M., Edden, R. A. E., Foley, A., Kennedy, D. N., Moore, C. M., & Frazier, J. A. (2015). Relationship among Glutamine, γ-Aminobutyric Acid, and Social Cognition in Autism Spectrum Disorders. Journal of Child and Adolescent Psychopharmacology, 25(4), 314–322. https://doi.org/10.1089/cap.2014.0112

4．Drenthen, G. S., Barendse, E. M., Aldenkamp, A. P., van Veenendaal, T. M., Puts, N. A. J., Edden, R. A. E., Zinger, S., Thoonen, G., Hendriks, M. P. H., Kessels, R. P. C., & Jansen, J. F. A. (2016). Altered neurotransmitter metabolism in adolescents with high-functioning autism. Psychiatry Research: Neuroimaging, 256, 44–49. https://doi.org/10.1016/j.pscychresns.2016.09.007

5．Gaetz, W., Bloy, L., Wang, D. J., Port, R. G., Blaskey, L., Levy, S. E., & Roberts, T. P. (2014). GABA estimation in the brains of children on the autism spectrum: measurement precision and regional cortical variation. NeuroImage, 86, 1–9. https://doi.org/10.1016/j.neuroimage.2013.05.068

6．Fung, L. K., Flores, R. E., Gu, M., Sun, K. L., James, D., Schuck, R. K., Jo, B., Park, J. H., Lee, B. C., Jung, J. H., Kim, S. E., Saggar, M., Sacchet, M. D., Warnock, G., Khalighi, M. M., Spielman, D., Chin, F. T., & Hardan, A. Y. (2021). Thalamic and prefrontal GABA concentrations but not GABAA receptor densities are altered in high-functioning adults with autism spectrum disorder. Molecular Psychiatry, 26(5), 1634–1646. https://doi.org/10.1038/s41380-020-0756-y

7．Harada, M., Taki, M. M., Nose, A., Kubo, H., Mori, K., Nishitani, H., & Matsuda, T. (2011). Non-Invasive Evaluation of the GABAergic/Glutamatergic System in Autistic Patients Observed by MEGA-Editing Proton MR Spectroscopy Using a Clinical 3 Tesla Instrument. Journal of Autism and Developmental Disorders, 41(4), 447–454. https://doi.org/10.1007/s10803-010-1065-0

8．He, J. L., Oeltzschner, G., Mikkelsen, M., Deronda, A., Harris, A. D., Crocetti, D., Wodka, E. L., Mostofsky, S. H., Edden, R. A. E., & Puts, N. A. J. (2021). Region-specific elevations of glutamate + glutamine correlate with the sensory symptoms of autism spectrum disorders. Translational Psychiatry, 11(1), 411. <https://doi.org/10.1038/s41398-021-01525-1>

9．Hegarty, J. P., 2nd, Weber, D. J., Cirstea, C. M., & Beversdorf, D. Q. (2018). Cerebro-Cerebellar Functional Connectivity is Associated with Cerebellar Excitation-Inhibition Balance in Autism Spectrum Disorder. Journal of autism and developmental disorders, 48(10), 3460–3473. https://doi.org/10.1007/s10803-018-3613-y

10．Horder, J., Petrinovic, M. M., Mendez, M. A., Bruns, A., Takumi, T., Spooren, W., Barker, G. J., Künnecke, B., & Murphy, D. G. (2018). Glutamate and GABA in autism spectrum disorder—A translational magnetic resonance spectroscopy study in man and rodent models. Translational Psychiatry, 8(1), 106. https://doi.org/10.1038/s41398-018-0155-1

11．Ito, H., Mori, K., Harada, M., Hisaoka, S., Toda, Y., Mori, T., Goji, A., Abe, Y., Miyazaki, M., & Kagami, S. (2017). A Proton Magnetic Resonance Spectroscopic Study in Autism Spectrum Disorder Using a 3-Tesla Clinical Magnetic Resonance Imaging (MRI) System: The Anterior Cingulate Cortex and the Left Cerebellum. Journal of Child Neurology, 32(8), 731–739. https://doi.org/10.1177/0883073817702981

12．Kolodny, T., Schallmo, M., Gerdts, J., Edden, R. A. E., Bernier, R. A., & Murray, S. O. (2020). Concentrations of Cortical GABA and Glutamate in Young Adults With Autism Spectrum Disorder. Autism Research, 13(7), 1111–1129. https://doi.org/10.1002/aur.2300

13．Kubas, B., Kułak, W., Sobaniec, W., Tarasow, E., Łebkowska, U., & Walecki, J. (2012). Metabolite alterations in autistic children: A 1H MR spectroscopy study. Advances in Medical Sciences, 57(1), 152–156. https://doi.org/10.2478/v10039-012-0014-x

14．Maier, S., Düppers, A. L., Runge, K., Dacko, M., Lange, T., Fangmeier, T., Riedel, A., Ebert, D., Endres, D., Domschke, K., Perlov, E., Nickel, K., & Tebartz van Elst, L. (2022). Increased prefrontal GABA concentrations in adults with autism spectrum disorders. Autism Research, 15(7), 1222–1236. https://doi.org/10.1002/aur.2740

15．Pretzsch, C. M., Freyberg, J., Voinescu, B., Lythgoe, D., Horder, J., Mendez, M. A., Wichers, R., Ajram, L., Ivin, G., Heasman, M., Edden, R. A. E., Williams, S., Murphy, D. G. M., Daly, E., & McAlonan, G. M. (2019). Effects of cannabidiol on brain excitation and inhibition systems; a randomised placebo-controlled single dose trial during magnetic resonance spectroscopy in adults with and without autism spectrum disorder. Neuropsychopharmacology, 44(8), 1398–1405. https://doi.org/10.1038/s41386-019-0333-8

16．Robertson, C. E., Ratai, E.-M., & Kanwisher, N. (2016). Reduced GABAergic Action in the Autistic Brain. Current Biology, 26(1), 80–85. https://doi.org/10.1016/j.cub.2015.11.019

17．Rojas, D. C., Singel, D., Steinmetz, S., Hepburn, S., & Brown, M. S. (2014). Decreased left perisylvian GABA concentration in children with autism and unaffected siblings. NeuroImage, 86, 28–34. https://doi.org/10.1016/j.neuroimage.2013.01.045

18．Siegel-Ramsay, J. E., Romaniuk, L., Whalley, H. C., Roberts, N., Branigan, H., Stanfield, A. C., Lawrie, S. M., & Dauvermann, M. R. (2021). Glutamate and functional connectivity—Support for the excitatory-inhibitory imbalance hypothesis in autism spectrum disorders. Psychiatry Research: Neuroimaging, 313, 111302. https://doi.org/10.1016/j.pscychresns.2021.111302

19．Wood, E. T., Cummings, K. K., Jung, J., Patterson, G., Okada, N., Guo, J., O’Neill, J., Dapretto, M., Bookheimer, S. Y., & Green, S. A. (2021). Sensory over-responsivity is related to GABAergic inhibition in thalamocortical circuits. Translational Psychiatry, 11(1), 39. https://doi.org/10.1038/s41398-020-01154-0

Psychotic disorder

Schizophrenia (SZ)(N=28)

1. Bojesen, K. B., Ebdrup, B. H., Jessen, K., Sigvard, A., Tangmose, K., Edden, R. A. E., Larsson, H. B. W., Rostrup, E., Broberg, B. V., & Glenthøj, B. Y. (2020). Treatment response after 6 and 26 weeks is related to baseline glutamate and GABA levels in antipsychotic-naïve patients with psychosis. Psychological Medicine, 50(13), 2182–2193. https://doi.org/10.1017/S0033291719002277
2. Brandt, A. S., Unschuld, P. G., Pradhan, S., Lim, I. A. L., Churchill, G., Harris, A. D., Hua, J., Barker, P. B., Ross, C. A., van Zijl, P. C. M., Edden, R. A. E., & Margolis, R. L. (2016). Age-related changes in anterior cingulate cortex glutamate in schizophrenia: A 1H MRS Study at 7Tesla. Schizophrenia Research, 172(1–3), 101–105. https://doi.org/10.1016/j.schres.2016.02.017
3. Chen, T., Wang, Y., Zhang, J., Wang, Z., Xu, J., Li, Y., Yang, Z., & Liu, D. (2017). Abnormal Concentration of GABA and Glutamate in The Prefrontal Cortex in Schizophrenia.-An in Vivo 1H-MRS Study. 29(5).
4. Chiu, P. W., Lui, S. S. Y., Hung, K. S. Y., Chan, R. C. K., Chan, Q., Sham, P. C., Cheung, E. F. C., & Mak, H. K. F. (2018). In vivo gamma-aminobutyric acid and glutamate levels in people with first-episode schizophrenia: A proton magnetic resonance spectroscopy study. Schizophrenia Research, 193, 295–303. https://doi.org/10.1016/j.schres.2017.07.021
5. de la Fuente-Sandoval, C., León-Ortiz, P., Azcárraga, M., Stephano, S., Favila, R., Díaz-Galvis, L., Alvarado-Alanis, P., Ramírez-Bermúdez, J., & Graff-Guerrero, A. (2013). Glutamate Levels in the Associative Striatum Before and After 4 Weeks of Antipsychotic Treatment in First-Episode Psychosis: A Longitudinal Proton Magnetic Resonance Spectroscopy Study. JAMA Psychiatry, 70(10), 1057. https://doi.org/10.1001/jamapsychiatry.2013.289
6. de la Fuente-Sandoval, C., Reyes-Madrigal, F., Mao, X., León-Ortiz, P., Rodríguez-Mayoral, O., Jung-Cook, H., Solís-Vivanco, R., Graff-Guerrero, A., & Shungu, D. C. (2018). Prefrontal and Striatal Gamma-Aminobutyric Acid Levels and the Effect of Antipsychotic Treatment in First-Episode Psychosis Patients. Biological Psychiatry, 83(6), 475–483. https://doi.org/10.1016/j.biopsych.2017.09.028
7. Goto, N., Yoshimura, R., Moriya, J., Kakeda, S., Ueda, N., Ikenouchi-Sugita, A., Umene-Nakano, W., Hayashi, K., Oonari, N., Korogi, Y., & Nakamura, J. (2009). Reduction of brain γ-aminobutyric acid (GABA) concentrations in early-stage schizophrenia patients: 3T Proton MRS study. Schizophrenia Research, 112(1–3), 192–193. https://doi.org/10.1016/j.schres.2009.04.026
8. Grent-’t-Jong, T., Gross, J., Goense, J., Wibral, M., Gajwani, R., Gumley, A. I., Lawrie, S. M., Schwannauer, M., Schultze-Lutter, F., Navarro Schröder, T., Koethe, D., Leweke, F. M., Singer, W., & Uhlhaas, P. J. (2018). Resting-state gamma-band power alterations in schizophrenia reveal E/I-balance abnormalities across illness-stages. ELife, 7, e37799. https://doi.org/10.7554/eLife.37799
9. Kelemen, O., Kiss, I., Benedek, G., & Kéri, S. (2013). Perceptual and cognitive effects of antipsychotics in first-episode schizophrenia: The potential impact of GABA concentration in the visual cortex. Progress in Neuro-Psychopharmacology and Biological Psychiatry, 47, 13–19. https://doi.org/10.1016/j.pnpbp.2013.07.024
10. Mao, X. (2012). Elevated Prefrontal Cortex γ-Aminobutyric Acid and Glutamate-Glutamine Levels in Schizophrenia Measured In Vivo With Proton Magnetic Resonance Spectroscopy. Archives of General Psychiatry, 69(5), 449. https://doi.org/10.1001/archgenpsychiatry.2011.1519
11. Marenco, S., Meyer, C., Kuo, S., van der Veen, J. W., Shen, J., DeJong, K., Barnett, A. S., Apud, J. A., Dickinson, D., Weinberger, D. R., & Berman, K. F. (2016). Prefrontal GABA Levels Measured With Magnetic Resonance Spectroscopy in Patients With Psychosis and Unaffected Siblings. American Journal of Psychiatry, 173(5), 527–534. https://doi.org/10.1176/appi.ajp.2015.15020190
12. Marsman, A., Mandl, R. C. W., Klomp, D. W. J., Bohlken, M. M., Boer, V. O., Andreychenko, A., Cahn, W., Kahn, R. S., Luijten, P. R., & Hulshoff Pol, H. E. (2014). GABA and glutamate in schizophrenia: A 7 T 1H-MRS study. NeuroImage: Clinical, 6, 398–407. https://doi.org/10.1016/j.nicl.2014.10.005
13. Mcilwain, M. E., Anderson, V. M., Pillai, A., Kydd, R. R., & Russell, B. R. (2015). Glutamatergic Neurometabolites in Clozapine-Responsive and -Resistant Schizophrenia. International Journal of Neuropsychopharmacology, 18(6), pyu117. https://doi.org/10.1093/ijnp/pyu117
14. Öngür, D., Prescot, A. P., McCarthy, J., Cohen, B. M., & Renshaw, P. F. (2010). Elevated Gamma-Aminobutyric Acid Levels in Chronic Schizophrenia. Biological Psychiatry, 68(7), 667–670. https://doi.org/10.1016/j.biopsych.2010.05.016
15. Ragland, J. D., Maddock, R. J., Hurtado, M. Y., Tanase, C., Lesh, T. A., Niendam, T. A., Carter, C. S., & Ranganath, C. (2020). Disrupted GABAergic facilitation of working memory performance in people with schizophrenia. NeuroImage: Clinical, 25, 102127. https://doi.org/10.1016/j.nicl.2019.102127
16. Reid, M. A., Salibi, N., White, D. M., Gawne, T. J., Denney, T. S., & Lahti, A. C. (2019). 7T Proton Magnetic Resonance Spectroscopy of the Anterior Cingulate Cortex in First-Episode Schizophrenia. Schizophrenia Bulletin, 45(1), 180–189. https://doi.org/10.1093/schbul/sbx190
17. Rowland, L. M., Kontson, K., West, J., Edden, R. A., Zhu, H., Wijtenburg, S. A., Holcomb, H. H., & Barker, P. B. (2013). In Vivo Measurements of Glutamate, GABA, and NAAG in Schizophrenia. Schizophrenia Bulletin, 39(5), 1096–1104. https://doi.org/10.1093/schbul/sbs092
18. Rowland, L. M., Summerfelt, A., Wijtenburg, S. A., Du, X., Chiappelli, J. J., Krishna, N., West, J., Muellerklein, F., Kochunov, P., & Hong, L. E. (2016). Frontal Glutamate and γ-Aminobutyric Acid Levels and Their Associations With Mismatch Negativity and Digit Sequencing Task Performance in Schizophrenia. JAMA Psychiatry, 73(2), 166. https://doi.org/10.1001/jamapsychiatry.2015.2680
19. Shukla, D. K., Wijtenburg, S. A., Chen, H., Chiappelli, J. J., Kochunov, P., Hong, L. E., & Rowland, L. M. (2019). Anterior Cingulate Glutamate and GABA Associations on Functional Connectivity in Schizophrenia. Schizophrenia Bulletin, 45(3), 647–658. https://doi.org/10.1093/schbul/sby075
20. Sivaraman, S., Kraguljac, N. V., White, D. M., Morgan, C. J., Gonzales, S. S., & Lahti, A. C. (2018). Neurometabolic abnormalities in the associative striatum in antipsychotic-naïve first episode psychosis patients. Psychiatry Research: Neuroimaging, 281, 101–106. https://doi.org/10.1016/j.pscychresns.2018.06.003
21. Stan, A. D., Ghose, S., Zhao, C., Hulsey, K., Mihalakos, P., Yanagi, M., Morris, S. U., Bartko, J. J., Choi, C., & Tamminga, C. A. (2015). Magnetic resonance spectroscopy and tissue protein concentrations together suggest lower glutamate signaling in dentate gyrus in schizophrenia. Molecular Psychiatry, 20(4), 433–439. https://doi.org/10.1038/mp.2014.54
22. Tarumi, R., Tsugawa, S., Noda, Y., Plitman, E., Honda, S., Matsushita, K., Chavez, S., Sawada, K., Wada, M., Matsui, M., Fujii, S., Miyazaki, T., Chakravarty, M. M., Uchida, H., Remington, G., Graff-Guerrero, A., Mimura, M., & Nakajima, S. (2020). Levels of glutamatergic neurometabolites in patients with severe treatment-resistant schizophrenia: A proton magnetic resonance spectroscopy study. Neuropsychopharmacology, 45(4), 632–640. https://doi.org/10.1038/s41386-019-0589-z
23. Tayoshi, S., Nakataki, M., Sumitani, S., Taniguchi, K., Shibuya-Tayoshi, S., Numata, S., Iga, J., Ueno, S., Harada, M., & Ohmori, T. (2010). GABA concentration in schizophrenia patients and the effects of antipsychotic medication: A proton magnetic resonance spectroscopy study. Schizophrenia Research, 117(1), 83–91. https://doi.org/10.1016/j.schres.2009.11.011
24. Tayoshi, S., Sumitani, S., Taniguchi, K., Shibuya-Tayoshi, S., Numata, S., Iga, J., Nakataki, M., Ueno, S., Harada, M., & Ohmori, T. (2009). Metabolite changes and gender differences in schizophrenia using 3-Tesla proton magnetic resonance spectroscopy (1H-MRS). Schizophrenia Research, 108(1–3), 69–77. https://doi.org/10.1016/j.schres.2008.11.014
25. Thakkar, K. N., Rösler, L., Wijnen, J. P., Boer, V. O., Klomp, D. W. J., Cahn, W., Kahn, R. S., & Neggers, S. F. W. (2017). 7T Proton Magnetic Resonance Spectroscopy of Gamma-Aminobutyric Acid, Glutamate, and Glutamine Reveals Altered Concentrations in Patients With Schizophrenia and Healthy Siblings. Biological Psychiatry, 81(6), 525–535. https://doi.org/10.1016/j.biopsych.2016.04.007
26. Wang, A. M., Pradhan, S., Coughlin, J. M., Trivedi, A., DuBois, S. L., Crawford, J. L., Sedlak, T. W., Nucifora, F. C., Nestadt, G., Nucifora, L. G., Schretlen, D. J., Sawa, A., & Barker, P. B. (2019). Assessing Brain Metabolism With 7-T Proton Magnetic Resonance Spectroscopy in Patients With First-Episode Psychosis. JAMA Psychiatry, 76(3), 314. https://doi.org/10.1001/jamapsychiatry.2018.3637
27. White, D. M., Kraguljac, N. V., Reid, M. A., & Lahti, A. C. (2015). Contribution of substantia nigra glutamate to prediction error signals in schizophrenia: A combined magnetic resonance spectroscopy/functional imaging study. Npj Schizophrenia, 1(1), 14001. https://doi.org/10.1038/npjschz.2014.1
28. Wijtenburg, S. A., Wang, M., Korenic, S. A., Chen, S., Barker, P. B., & Rowland, L. M. (2021). Metabolite Alterations in Adults With Schizophrenia, First Degree Relatives, and Healthy Controls: A Multi-Region 7T MRS Study. Frontiers in Psychiatry, 12, 656459. https://doi.org/10.3389/fpsyt.2021.656459
29. Xin, L., Mekle, R., Fournier, M., Baumann, P. S., Ferrari, C., Alameda, L., Jenni, R., Lu, H., Schaller, B., Cuenod, M., Conus, P., Gruetter, R., & Do, K. Q. (2016). Genetic Polymorphism Associated Prefrontal Glutathione and Its Coupling With Brain Glutamate and Peripheral Redox Status in Early Psychosis. Schizophrenia Bulletin, 42(5), 1185–1196. https://doi.org/10.1093/schbul/sbw038
30. Yoon, J. H., Maddock, R. J., Rokem, A., Silver, M. A., Minzenberg, M. J., Ragland, J. D., & Carter, C. S. (2010). GABA Concentration Is Reduced in Visual Cortex in Schizophrenia and Correlates with Orientation-Specific Surround Suppression. The Journal of Neuroscience, 30(10), 3777–3781. https://doi.org/10.1523/JNEUROSCI.6158-09.2010

Other

Alcohol use disorder (N=7)

1. Behar, K. L., Rothman, D. L., Petersen, K. F., Hooten, M., Delaney, R., Petroff, O. A. C., Shulman, G. I., Navarro, V., Petrakis, I. L., Charney, D. S., & Krystal, J. H. (1999). Preliminary Evidence of Low Cortical GABA Levels in Localized 1 H-MR Spectra of Alcohol-Dependent and Hepatic Encephalopathy Patients. American Journal of Psychiatry, 156(6), 952–954. https://doi.org/10.1176/ajp.156.6.952
2. Hermann, D., Weber-Fahr, W., Sartorius, A., Hoerst, M., Frischknecht, U., Tunc-Skarka, N., Perreau-Lenz, S., Hansson, A. C., Krumm, B., Kiefer, F., Spanagel, R., Mann, K., Ende, G., & Sommer, W. H. (2012). Translational Magnetic Resonance Spectroscopy Reveals Excessive Central Glutamate Levels During Alcohol Withdrawal in Humans and Rats. Biological Psychiatry, 71(11), 1015–1021. https://doi.org/10.1016/j.biopsych.2011.07.034
3. Lee, E., Jang, D.-P., Kim, J.-J., An, S. K., Park, S., Kim, I.-Y., Kim, S. I., Yoon, K.-J., & Namkoong, K. (2007). Alteration of brain metabolites in young alcoholics without structuralchanges.NeuroReport,18(14),1511–1514. https://doi.org/10.1097/WNR.0b013e32

82ef7625

1. Mon, A., Durazzo, T. C., & Meyerhoff, D. J. (2012). Glutamate, GABA, and other cortical metabolite concentrations during early abstinence from alcohol and their associations with neurocognitive changes. Drug and Alcohol Dependence, 125(1–2), 27–36. https://doi.org/10.1016/j.drugalcdep.2012.03.012
2. Prisciandaro, J. J., Schacht, J. P., Prescot, A. P., Brenner, H. M., Renshaw, P. F., Brown, T. R., & Anton, R. F. (2020). Intraindividual changes in brain GABA, glutamate, and glutamine during monitored abstinence from alcohol in treatment‐naive individuals with alcohol use disorder. Addiction Biology, 25(6). https://doi.org/10.1111/adb.12810
3. Thoma, R., Mullins, P., Ruhl, D., Monnig, M., Yeo, R. A., Caprihan, A., Bogenschutz, M., Lysne, P., Tonigan, S., Kalyanam, R., & Gasparovic, C. (2011). Perturbation of the Glutamate–Glutamine System in Alcohol Dependence and Remission. Neuropsychopharmacology, 36(7), 1359–1365. https://doi.org/10.1038/npp.2011.20
4. Wang, G., Weber-Fahr, W., Frischknecht, U., Hermann, D., Kiefer, F., Ende, G., & Sack, M. (2021). Cortical Glutamate and GABA Changes During Early Abstinence in Alcohol Dependence and Their Associations With Benzodiazepine Medication. Frontiers in Psychiatry, 12, 656468. https://doi.org/10.3389/fpsyt.2021.656468
